# Physical activity and incident type 2 diabetes mellitus: a systematic review and dose–response meta-analysis of prospective cohort studies

**DOI:** 10.1007/s00125-016-4079-0

**Published:** 2016-10-17

**Authors:** Andrea D. Smith, Alessio Crippa, James Woodcock, Søren Brage

**Affiliations:** 1 Health Behaviour Research Centre, Department of Epidemiology and Public Health, University College London, 1-19 Torrington Place, London, WC1E 6BT UK; 2 Institute of Public Health and Primary Care, University of Cambridge, Cambridge, UK; 3 Institute of Environmental Medicine, Karolinska Institutet, Solna, Sweden; 4 UKCRC Centre for Diet and Activity Research (CEDAR), University of Cambridge, Institute of Metabolic Science, Cambridge, UK; 5 MRC Epidemiology Unit, University of Cambridge, Institute of Metabolic Science, Cambridge, UK

**Keywords:** Cohort studies, Dose–response, Meta-analysis, Physical activity, Systematic review, Type 2 diabetes

## Abstract

**Aims/hypothesis:**

Inverse associations between physical activity (PA) and type 2 diabetes mellitus are well known. However, the shape of the dose–response relationship is still uncertain. This review synthesises results from longitudinal studies in general populations and uses non-linear models of the association between PA and incident type 2 diabetes.

**Methods:**

A systematic literature search identified 28 prospective studies on leisure-time PA (LTPA) or total PA and risk of type 2 diabetes. PA exposures were converted into metabolic equivalent of task (MET) h/week and marginal MET (MMET) h/week, a measure only considering energy expended above resting metabolic rate. Restricted cubic splines were used to model the exposure–disease relationship.

**Results:**

Our results suggest an overall non-linear relationship; using the cubic spline model we found a risk reduction of 26% (95% CI 20%, 31%) for type 2 diabetes among those who achieved 11.25 MET h/week (equivalent to 150 min/week of moderate activity) relative to inactive individuals. Achieving twice this amount of PA was associated with a risk reduction of 36% (95% CI 27%, 46%), with further reductions at higher doses (60 MET h/week, risk reduction of 53%). Results for the MMET h/week dose–response curve were similar for moderate intensity PA, but benefits were greater for higher intensity PA and smaller for lower intensity activity.

**Conclusions/interpretation:**

Higher levels of LTPA were associated with substantially lower incidence of type 2 diabetes in the general population. The relationship between LTPA and type 2 diabetes was curvilinear; the greatest relative benefits are achieved at low levels of activity, but additional benefits can be realised at exposures considerably higher than those prescribed by public health recommendations.

**Electronic supplementary material:**

The online version of this article (doi:10.1007/s00125-016-4079-0) contains peer-reviewed but unedited supplementary material, which is available to authorised users.

## Introduction

High fasting plasma glucose was recently ranked as the fifth leading risk for death [1] and 6.8% of global excess mortality was attributed to diabetes [2]. Prevalence of this metabolic disorder is predicted to reach nearly 600 million cases by 2035 [3], posing both a substantial morbidity and mortality burden and a large financial cost on individuals and healthcare systems [4, 5].

Evidence on the effects of physical activity (PA) on risk of diabetes arises from interventional [6–9] and observational studies [10–14]. Prevention trials conducted in patients with impaired glucose tolerance provide some understanding of the extent to which PA may confer a preventive effect on progression to type 2 diabetes in high-risk populations [6–9, 15]. However, the majority of these studies include both diet and PA interventions, and isolation of the impact of PA itself is rarely possible. It is also difficult to evaluate the benefit of the whole PA exposure continuum from trials, as most intervention studies focus on shifting participants’ behaviours towards the recommended levels of exercise rather than assessing the benefits of changes at the lowest ends of the normal PA spectrum, or the additional benefits gained at the highest level. Therefore, although associated with a higher risk of confounding, evidence from cohort studies in the general population can provide complementary evidence of the dose–response relationship between PA and diabetes, independent of diet.

Public health guidelines [16, 17] recommend a minimum of 150 min of moderate to vigorous PA (MVPA) or 75 min vigorous PA (VPA) a week to maintain general health. Self-report data suggest that around a third of adults globally are not meeting these targets [18]. A fundamental consideration in the formulation of PA guidelines, however, is the nature of the dose–response relationship between PA and non-communicable disease incidence.

Dose–response curves for PA and health outcomes, ranging from cardiovascular disease to all-cause mortality, suggest a non-linear dose–response shape [19–24], often with large gains when low activity is compared with completely sedentary but much smaller additional benefits beyond that. A recent review suggested a non-linear relationship between PA and diabetes. However, it found differently shaped dose–response curves based on the different ways in which PA was reported in the original studies [25]. Each of the dose–response analyses only included a small portion of the total studies available in this area of research, owing to a lack of data harmonisation and leaving considerable uncertainty about the relative risk for any given exposure since not all of the evidence could be considered.

Providing quantitative estimates regarding the dose–response relationship is essential for approximating how changes in levels of PA in the general population would impact disease incidence, and would support more nuanced guidance to the public and evidence-based dialogue in clinical settings.

Calculating the dose of PA is associated with considerable uncertainty and can be achieved using a variety of methods. In deciding how to equate activities of varying intensity, one issue is whether to include the resting metabolic rate. In this review we investigate the dose–response relationship between PA and type 2 diabetes via a systematic review and dose–response meta-analysis. We report results quantifying PA dose, both via inclusion and exclusion of the resting metabolic rate in the summation of PA volume.

## Methods

### Search strategy

**Fig. 1:**
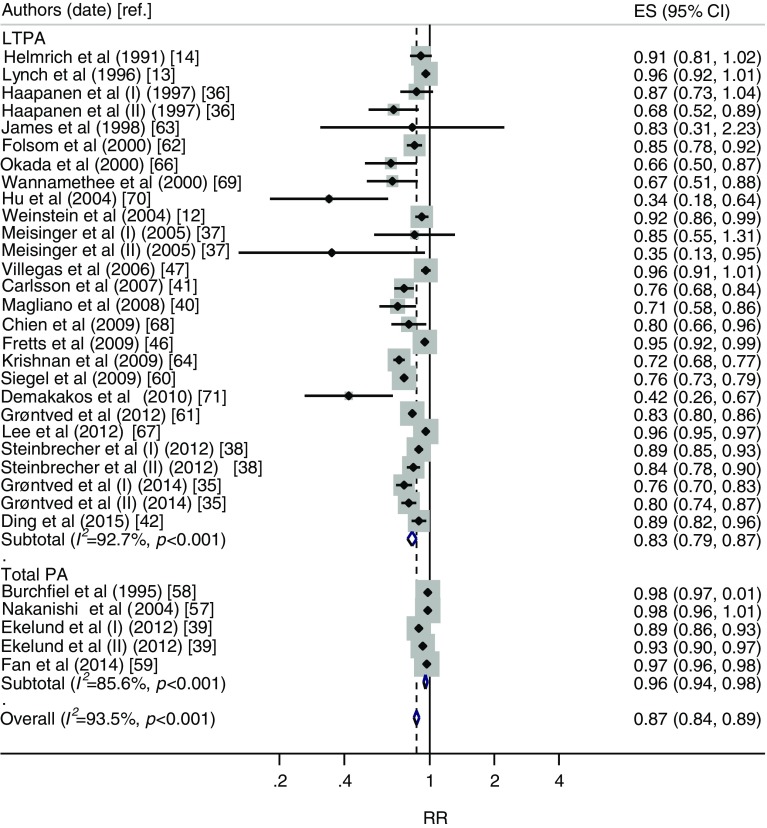
Forest plot of the study-specific RRs for type 2 diabetes for every 10 MET h/week exposure of PA, sorted by PA domain and publication year. Study-specific estimates obtained by a generalised least squares regression assuming a linear relationship of the RR to the referent in a random-effects model. Referents for PA were the individuals reporting no or lowest level of PA within the specific study. (I)/(II) indicate subcohorts with independently reported risk estimates for type 2 diabetes. The black midline indicates the line of no effect. The diamond indicates the pooled (subgroup) estimate. Grey boxes are relative to study size and the black vertical lines indicate 95% CIs around the effect size estimate

PubMed and EMBASE were searched for prospective cohort studies on the association between PA and type 2 diabetes using a combination of medical subject heading (MeSH) and indexed terms (details in electronic supplementary material [ESM] Fig. 1). Search filters for observational studies were applied to refine the search output. The reference list of past systematic reviews were manually searched for further studies [26–32]. No restrictions on date of publication were set and new results were included up until December 2015.

### Eligibility criteria

Prospective studies were included if they: (1) followed a cohort of adults; (2) excluded individuals with type 2 diabetes at baseline; (3) ascertained levels of leisure-time PA (LTPA) or total PA at baseline; and (4) reported RRs, ORs or HRs for incidence of type 2 diabetes. Exclusion criteria were: (1) studies which reported insufficient detail of PA assessment to estimate PA dose in metabolic equivalent of task (MET) h/week; (2) studies using measures of fitness as the exposure; (3) studies reporting PA as a dichotomous variable; and (4) duplicate data.

Two researchers (ADS and BR-S) screened titles and abstracts for eligibility according to the pre-specified criteria. When eligibility was ambiguous, the full text was retrieved. To ensure no duplicate data were included, cohort name, recruitment periods or protocols were compared, and only the most complete publication was included. A third researcher (O. Olayinka, London School of Hygiene and Tropical Medicine, London, UK) assessed the identified articles and any disagreements were discussed until consensus was reached. A breakdown of the literature search is shown in ESM Fig. 2.

### Data extraction and exposure harmonisation

Data were extracted (by ADS) from eligible studies on first author, publication date, geographical location, cohort size, sex and age characteristics, cumulative incidence or incidence rate of type 2 diabetes, case count per category of PA exposure, total persons or person-years per PA category, method and unit of PA assessment, reported levels of PA exposure, ORs/RRs/HRs for type 2 diabetes with 95% CIs for each PA category, and covariates for which the analyses were adjusted. Overall study quality score was derived using the Newcastle Ottawa Scale (NOS); inter-rater reliability (between ADS and O. Olayinka) was 86% (full NOS results are shown in ESM Table 1).

In prospective studies where HRs or ORs for type 2 diabetes were reported, we assumed these approximated the RR [33]. We pooled the most adjusted risk estimates both including and excluding adjustment for BMI. Initially we harmonised group-level exposure estimates to the common unit of MET h/week, thereby allowing integration of activities differing in intensity and duration amassed over the course of a week. For the assignment of specific intensities to categories of PA exposure, average intensity of MVPA and VPA was defined as 4.5 and 8 METs (or 3.5 and 7 marginal METs [MMETs]), respectively [34]. Studies reporting data independently for men and women [35–39] or for multiple cohorts within a study [35] were treated as separate observations. Studies reporting risk estimates relative to the highest category of PA were re-calculated to set the lowest PA [36, 40–42] category as the referent [43].

When not directly reported, classic PA volume (MET h/week) was calculated by multiplication of the median or mid-point duration of the reported category with its assigned gross MET value. Open-ended categories for average LTPA duration were converted to point estimates by assuming that the median of the open-ended category was equidistant from the lower category boundary as half the interval width in the neighbouring category [44]. For one study that reported PA as PA level (PAL, a measure of energy expenditure expressed as a multiple of 24 h resting metabolic rate), an approximation of LTPA MET h/week was performed using descriptions of typical PA levels for each category [45]. If PA was reported only as frequency of sessions per week, a single session was assumed to consist of 45 min in the main analysis with an assumption of 30 min tested in sensitivity analysis. Likewise, if only average duration for PA (e.g. walking, cycling) was reported, we assumed this was undertaken at an intensity of 4.5 METs. Marginalised PA volume (MMET h/week) was calculated by discounting the resting metabolic rate of 1 MET in the quantification of PA intensity. An overview of dose assignment calculations is shown in ESM Table 2. For summary data, we subtracted 1 MET h from each 1 h increment over which total reported activity was performed. When the required data were not reported in the original articles we emailed authors from the identified cohorts to acquire further details, e.g. on duration of PA and number of type 2 diabetes cases for each PA exposure category. Following correspondence, updated follow-up data [11, 13] and further details on PA behaviour [11, 38, 46, 47] were obtained.

### Statistical analysis

Generalised least-squares (GLS) regression was performed to estimate study-specific dose–response curves. GLS regression estimates the linear dose–response coefficients taking into account the covariance for each exposure category within each study, as they are estimated relative to a common referent PA exposure category [48, 49]. Study-specific dose–response coefficients were pooled using the DerSimonian–Laird estimator in a random-effects model [50]. First, a linear association was assumed; study-specific RR estimates were calculated per 10 MET h/week increment and subsequently pooled. Two cohorts [51, 52] did not provide sufficient data to be included in this model. However, variance-weighted least-squares regression analysis was used to estimate linear associations for both of these studies, allowing us to quantify the influence of excluding these on the overall effect estimates.

Sensitivity analyses were conducted by consecutive removal of individual studies from the summary risk estimate and via restriction to high-quality studies. The impact of duration and intensity assumptions (when necessary) was assessed by applying lower values. Subgroup analysis by sex, study location, cohort size and follow-up time was undertaken. Mediation by BMI was explored according to the degree of adjustment (BMI adjusted vs non-BMI adjusted) and participant obesity (BMI < 30 vs > 30 kg/m^2^). To further reduce heterogeneity, we separately pooled risk estimates that either focused on LTPA or the more inclusive measures of total PA. Significance of subgroup and sensitivity analysis was judged by the *p* value for heterogeneity [53].

In addition, we examined possible non-linear associations by modelling PA using restricted cubic spline with three knots located at the 25th, 50th and 75th percentiles of the distribution. Only studies reporting risk estimates for at least three PA exposure levels for incident type 2 diabetes [54] were included in this analysis. Departure from linearity of the final cubic spline model was assessed using the Wald test for non-linearity [55].

Publication bias was investigated by funnel plot and Egger’s test for asymmetry. All reported *p* values were two sided. All analyses were performed using Stata 13.1 (Stata Corp, College Station, TX, USA). Interactive dose–response curves were visualised using R (R Foundation for Statistical Computing, Vienna, Austria) [56].

## Results

### Literature search

In total, 28 eligible cohort studies were identified which returned a total of 32 independent observations on PA and incidence of type 2 diabetes. The majority of studies (24 cohorts) yielded information on the association between LTPA and type 2 diabetes (28 observations), while four cohorts [39, 57–59] reported findings on total PA. Overall, this review includes 1,261,991 individuals and 84,134 incident cases of type 2 diabetes.

### Study characteristics

Cohort size ranged from 916 to 675,496 people, with cumulative type 2 diabetes incidence ranging from 1.6% [42] to 27.5% [46]. Follow-up time varied from 3 [42] to 23.1 [60] years. Twelve studies were conducted in the USA [12, 14, 35, 38, 46, 58, 60–65], six in Asia [47, 57, 59, 66–68], two in Australia [40, 42] and eight across Europe [13, 36, 37, 39, 41, 69–71]. All cohorts relied on self-reported PA collected using questionnaires or by interview, apart from one study in Hawaiians [58]. A descriptive summary of the cohort characteristics can be found in Table 1.

**Table 1:** Summary of the characteristics of 28 prospective cohort studies that investigate the association between levels of PA and incident type 2 diabetes, identified in the systematic literature search

Study	Country; study name	Cohort size	Sex	Age at baseline (years)	Follow-up (years)	% Cumulative incidence (cases/cohort)	PA unit	PA assessment (PA dose in MET h/week^a^)	Reported OR/RR/HR (95% CI)	Adjustments
Helmrich et al, 1991 [14]	USA; University of Pennsylvania Alumni	5990	M	39–68	14	3.4 (202/5990)	Weekly EE for LTPA	Weekly total EE for LTPA in 500 kcal (2092 kJ) increments: • <500 (3.3) • 500–999 (9.5) • 1000–1499 (16.4) • 1500–1999 (23.0) • 2000–2499 (29.5) • 2500–2999 (36.1) • 3000–3499 (42.7) • ≥3000 (49.2)	RR for T2D relative to most inactive group: 1.00 0.94 (0.9, 0.98) 0.79 0.78 0.68 0.90 0.86 0.52	Age
Burchfiel et al, 1995 [58]	USA; Honolulu Heart Program^b^	6815	M	45–68	6	5.7 (391/6815)	H/week in each of five activity levels (multiplied by a weight based on mean oxygen consumption required to perform the activities of the category)	Composite score based on 24 h PA dose calculated by summing the hours spent in each activity intensity level and multiplying by a respective weight factor and categorised into quintiles: • 24.1–29.0 (129.9) • 29.1–30.7 (153.3) • 30.8–33.2 (168.0) • 33.3–36.2 (187.3) • 36.3–65.5 (300.3)	OR for T2D relative to most inactive group: 1.00 0.86 (0.64, 1.16) 0.81 (0.60, 1.09) 0.72 (0.53, 1.03) 0.47 (0.33, 0.67)	Age
Lynch et al, 1996 [13]	Finland; Kuopio Ischaemic Heart Disease Risk Factor Study^b^	2682	M	42–60	18	23.9 (640/2682)	Frequency/month; intensity was estimated on a scale of 1 (lowest) –3 (highest)	1 year retrospective leisure time physical activity assessment of 15 common PA types: • low PA (4.1) • PA below an intensity of 5.5 MET but >2 h/week (36.1) • PA (>5.5 MET intensity) >40 min/week (46.7)	PA (>5.5 MET intensity) >40 min/week had an OR of 0.83 (0.63, 1.10) for T2D compared with participants reporting less duration/intensity of PA The OR for T2D observed for PA below an intensity of 5.5 MET but >2 h/week was 0.83 (0.66,1.03)	Age, fasting baseline glucose, serum triacylglycerol, BP, parental history of diabetes, alcohol consumption, BMI
Haapanen et al, 1997 [36]	Finland; North-Eastern Finnish Adult cohort (I)	1340	M	35–63^c^	10	4.8 (64/1340)	LTPA EE/week Frequency of vigorous PA/week	Weekly LTPA EE (kcal) categories for men: • low: 0–1100 (6.8) • moderate: 1101–1900 (18.5) • high: >1900 (28.3) Participants were asked to report average frequency of vigorous activity (≥6 MET) as: • ≥1/week • <1/week	RR for T2D relative to most inactive group: 1.00 1.54 (0.83, 2.84) 1.63 (0.92, 2.88)	Age
Haapanen et al, 1997 [36]	Finland; North-Eastern Finnish Adult cohort (II)	1500	F	35–63^c^	10	3.6 (54/1500)	LTPA EE/week Frequency of vigorous PA/week	Weekly LTPA EE (kcal) categories for women: • low: 0–900 (6.9) • moderate: 901–1500 (18.3) • high: >1500 (25.7) Participants were asked to report average frequency of vigorous activity (≥6 MET) as: • ≥1/week • <1/week	RR for T2D relative to most inactive group: 1.00 2.64 (1.28, 5.44) 2.23 (0.95, 5.23)	Age
James et al, 1998 [63]	USA; Pitt County Study	916	M/F	25–55	5	8.5 (78/916)	Physical activity index based on frequency of physical activity enough to work up a sweat and result in heavy breathing Frequency of strenuous work/exercise >20 min at a time	Four categories of LTPA level defined as: • ‘inactive’ = individuals who did not report any strenuous work/exercise, walking, home maintenance or gardening in a week. (0) • ‘low’ = some home maintenance work (>15 min) or gardening during an average week (1.1) • ‘moderate’ = some strenuous work/exercise but not >3 times/week at 20 min/session (4.5) • ‘strenuous’ = strenuous exercise/work >3/week and >20 min at a time (7.9)	OR for T2D relative to most inactive group: 1.00 0.51 (0.20, 1.28) 0.35 (0.12, 0.98) 0.65 (0.26, 1.63)	Age, sex, education, WHR, BMI
Folsom et al, 2000 [62]	USA; Iowa Women’s Health Study	34,257	F	55–69	12	5.8 (1997/34,257)	Frequency/week	Initial assessment of any habitual PA (Y/N) Participants reporting regular PA needed to specify frequency of moderate and vigorous PA (>6 MET) which was categorised into quartiles: • rare or never (0) • 1/week–few times/month (3.4) • 2–4/week (10.1) • >4/week (16.9)	RR for T2D relative to most inactive group: 1.00 0.80 (0.71, 0.90) 0.65 (0.58, 0.74) 0.51 (0.43, 0.59) BMI-adjusted RR for T2D relative to most inactive group: 1.00 0.90 (0.79, 1.01) 0.86 (0.76, 0.98) 0.73 (0.62, 0.85)	Age, education, smoking, alcohol intake, oestrogen replacement, energy intake, wholegrain intake, dietary score, family history of diabetes (+ BMI and WHR in further adjusted model)
Okada et al, 2000 [66]	Japan; Osaka Health Survey	6013	M	35–60	10	7.4 (444/6013)	Min/week during the week or weekend	Three categories of weekly LTPA: • ‘sedentary’: no regular exercise (0) • ‘moderate’: 1 h/week (5.1) • ‘vigorous’: ≥1 h/week exercise ‘enough to work up a sweat’ (15.2)	RR for T2D relative to most inactive group: 1.00 0.65 (0.45, 0.95) 0.52 (0.35, 0.79) BMI-adjusted RR for T2D relative to most inactive group: 1.00 0.80 (0.71, 0.99) 0.55 (0.34, 0.87)	Age, daily alcohol consumption, smoking habits, BP levels, parental history of T2D (+BMI in the BMI-adjusted model)
Wannamethee et al, 2000 [69]	UK; British Regional Heart Study	7735	M	40–59	16.8	2.5 (196/7735)	Weekly frequency of three intensity categories (combined to an overall PA score): (1) regular walking and cycling (2) recreational activity e.g. gardening or pleasure walking (3) sporting activity e.g. running, golf, swimming or tennis	A PA score was calculated depending on dose/type of regular exercise. Scores were categorised into five groups: • ‘inactive/occasional’ (irregular walking or recreational activity) (2.5) • ‘light’ (more frequent recreational activities or exercise <1/week, or regular walking + recreational activity) (3.4) • ‘moderate’ (frequent weekend recreational activities + regular walking, or sporting activity 1/week) (6.8) • ‘moderately vigorous’ (exercise 1/week or frequent cycling + recreational activities or walking or frequent sporting activities) (11.3) • ‘vigorous’ (very frequent exercise, or frequent exercise + recreational activities) (16.9)	RR for T2D relative to most inactive group: 1.00 0.65 (0.42, 1.00) 0.60 (0.38, 0.95) 0.42 (0.24, 0.72) 0.36 (0.21, 0.62) BMI-adjusted RR for T2D relative to most inactive group: 1.00 0.66 (0.42, 1.02) 0.65 (0.41, 1.03) 0.48 (0.28, 0.83) 0.46 (0.27, 0.79)	Age, smoking, alcohol, social class, pre-existing CHD (+BMI in the BMI-adjusted model)
Hu et al, 2004 [70]	Finland; Eastern and South-Western Finnish adults	4369	M/F	45–64	9.4	2.8 (120/4369)	Min/week	A simplified index for LTPA scores was derived and reported in three categories: • ‘low’ = light levels of occupational, commuting (<30 min) and inactive LTPA (0) • ‘moderate’ = 1 type of LTPA activity/week (3.4) • ‘high’ = 2 or 3 types of LTPA/week (8.4)	RR for T2D relative to most inactive group: 1.00 0.71 (0.46, 1.12) 0.32 (0.19, 0.56) BMI-adjusted RR for T2D relative to most inactive group: 1.00 0.85 (0.54, 1.34) 0.43 (0.25, 0.74)	Age, study year, sex, systolic BP, smoking, education (+ BMI in BMI-adjusted model)
Nakanishi et al, 2004 [57]	Japan; Japanese male office worker cohort	2924	M	35–59	7	5.8 (168/2924)	Daily EE for total PA	Quartiles of daily EE/kg for 20 activities: • <33.1 (119.1) • 33.1–36.7 (244.3) • 36.8–40.3 (269.9) • ≥40.4 (295.0)	RR for T2D relative to most inactive group: 1.00 0.65 (0.45, 0.95) 0.52 (0.35, 0.79) 0.27 (0.16, 0.45) BMI-adjusted RR for T2D relative to most inactive group: 1.00 0.76 (0.52, 1.11) 0.70 (0.46, 1.06) 0.41 (0.24, 0.70)	Age, family history of diabetes, alcohol consumption, cigarette smoking, weekly EE on PA, systolic BP, HDL-cholesterol and triacylglycerol at baseline (+BMI in the BMI-adjusted model)
Weinstein et al, 2004 [12]	USA; Women’s Health Study	37,878	F	55	6.9	3.6 (1361/37,878)	LTPA EE/week and min/week walking	EE/week (kcal) for LTPA in categories of: • 0–199 (1.4) • 200–599 (4.1) • 600–1499 (10.3) • >1500 (22.7)	HR for T2D relative to most inactive group: 1.00 0.78 (0.68, 0.90) 0.69 (0.59, 0.80) 0.74 (0.63, 0.88) BMI-adjusted HR for T2D relative to most inactive group: 1.00 0.91 (0.79, 1.06) 0.86 (0.74, 1.01) 0.82 (0.70, 0.97)	Age, family history of diabetes, smoking, alcohol, hormone therapy, hypertension, high cholesterol, dietary factors, randomised treatment group within the Women’s Health Study (+ BMI in the BMI-adjusted model)
Hsia et al, 2005 [52]	USA; Women’s Health Initiative^d^	87,907	F	63.8	5.1	2.6 (2271/87,907)	MET h/week	Categories of weekly MET h for total physical activity: • 0–2.2 (1.2) • 2.3–7.4 (4.9) • 7.5–13.9 (10.7) • 14.0–23.4 (18.7) • >23.4 (28.1)	RR for T2D relative to most inactive group: 1.00 0.91 (0.80, 1.03) 0.80 (0.70, 0.91) 0.86 (0.75, 0.99) 0.78 (0.67, 0.91)	Age, BMI alcohol, education, smoking, hypertension, hypercholesterolaemia, dietary fibre intake, per cent energy from carbohydrate
Meisinger et al, 2005 [37]	Germany; MONICA/KORA Augsburg Cohort Study (I)	4069	M	24–75^c^	7.4	3.6 (145/4069)	H/week Frequency/season (summer/winter)	Four categories of LTPA defined as: • ‘no activity’ = no sports in summer or winter (0) • ‘low activity’ = irregular, <1 h/week in at least one season (2.3) • ‘moderate’ = regular 1 h/week in at least one season (4.5) • ‘high’ = regular > 2 h/week in both seasons (11.3)	HR for T2D relative to most inactive group: 1.00 0.86 (0.57, 1.29) 0.73 (0.45, 1.20) 0.73 (0.45, 1.20)	Age, survey, actual hypertension, dyslipidaemia, parental history of diabetes, regular smoking, alcohol intake, education, BMI
Meisinger et al, 2005 [37]	Germany; MONICA/KORA Augsburg Cohort Study (II)	4034	F	24–75^c^	7.4	2 (82/4034)	H/week Frequency/season (summer/winter)	Four categories of LTPA defined as: • ‘no activity’ = no sports in summer or winter (0) • ‘low activity’ = irregular, <1 h/week in at least one season (2.3) • ‘moderate’ = regular 1 h/week in at least one season (4.5) • ‘high’ = regular, >2 h/week in both seasons (11.3)	HR for T2D relative to most inactive group: 1.00 0.85 (0.51, 1.41) 0.59 (0.31, 1.11) 0.21 (0.05, 0.86)	Age, survey, actual hypertension, dyslipidaemia, parental history of diabetes, regular smoking, alcohol intake, education, BMI
Villegas et al, 2006 [47]	China; Shanghai Women’s Health Study	70,658	F	40–70	4.6	2.8 (1973/70,658)	MET h/day/year	EE for retrospective regular LTPA during previous 5 years in MET h/day/year, DPA (including walking), CPA (bus or vehicle, walking or cycling <30 min/day or 30+ min/day) and EE in OPA (high/medium/low) LTPA h/day: • 0 (0) • <0.8 (3.6) • 0.8–1.99 (11.3) • >1.99 (27.0)	RR for T2D relative to most inactive group: 1.00 0.89 (0.76, 1.03) 0.99 (0.85, 1.15) 0.83 (0.70, 0.97)	Age, daily calories, education level, income level, occupation, smoking, alcohol, hypertension, chronic diseases
Carlsson et al, 2007 [41]	Sweden; Nord-Trøndelag Health Survey	38,800	M/F	≥20	11	1.9 (738/38,800)	Exercise frequency ranging from ‘never’ to ‘every day’	Frequency of weekly LTPA: • never (0) • <1×/week (1.7) • 1×/week (3.4) • 2–3×/week (8.4) • every day (23.6)	RR for T2D relative to most inactive group: 1.00 0.79 (0.64, 0.99) 0.61 (0.48, 0.77) 0.60 (0.48, 0.73) 0.49 (0.37, 0.66)	Sex, smoking, BMI
Magliano et al, 2008 [40]	Australia; The Australian Diabetes, Obesity and Lifestyle Study	5842	M/F	50.9	5	3.8 (224/5842)	Total LTPA time derived from sum of the time spent performing MVPA + double the time spent performing VPA in the previous week	Categories of weekly LTPA min/week: • inactive (0 min/week) • insufficient (1–49 min/week) (5.6) • sufficient (≥150 min/week) (14.6)	OR for T2D relative to most inactive group: 1.00 0.97 (0.58, 1.63) 0.64 (0.46, 0.89) Fully-adjusted OR for T2D relative to most inactive group: 1.00 0.86 (0.58, 1.27) 0.50 (0.35, 0.72)	Age, sex, waist circumference, smoking, education, hypertension, family history of diabetes, log FPG, hypertriacylglycerolaemia, low HDL-cholesterol and cholesterol
Chien et al, 2009 [68]	Taiwan; Chin-Shan community cardiovascular cohort study (CCCC)	1639	M/F	>35	9.02	19 (312/1639)	Sports, occupational and leisure PA frequency was rated on a 5-point Likert scale	Frequency of sports exercise was reported in quartiles corresponding to: • never (0) • rarely (2.3) • sometimes (6.8) • often (16.9)	RR for T2D relative to most inactive group: 1.00 0.83 (0.62, 1.12) 0.70 (0.52, 0.94) 0.74 (0.54, 1.03) BMI-adjusted RR for T2D relative to most inactive group: 1.00 0.82 (0.60, 1.12) 0.65 (0.47, 0.89) 0.68 (0.49, 0.95)	Age, sex, the metabolic syndrome, smoking, current alcohol drinking, marital status, education level, occupation, hypertension status, HDL-cholesterol, triacylglycerols, glucose levels, family history of diabetes, BMI
Fretts et al, 2009 [46]	USA; The Strong Heart Study	1651	M/F	45–74	10	27.5 (454/1651)	LTPA MET h/week Total PA MET h/week	LTPA MET h/week: • no activity • <8 MET h/week (3.5) • 8–24 MET h/week (15.3) • >24 MET h/week (64.2) Total PA MET h/week: • no activity • <30 MET h/week • 30–106 MET h/week • >106 MET h/week	OR for T2D relative to most inactive group: 1.00 1.04 (0.74, 1.47) 0.76 (0.55, 1.07) 0.68 (0.49, 0.95) BMI-adjusted RR for T2D relative to most inactive group: 1.00 1.09 (0.76, 1.56) 0.80 (0.56, 1.15) 0.75 (0.53, 1.00)	Age, study site, sex, education, cigarette smoking, alcohol use, family history of diabetes, systolic BP, diastolic BP, HDL-cholesterol, LDL-cholesterol, plasma fibrinogen, BMI
Krishnan et al, 2009 [64]	USA; Black Women’s Health Study	45,668	F	21–69	10	6.4 (2928/45,668)	H/week spent on VPA (e.g. running, swimming), walking for exercise and walking to and from work	MVPA was reported in categories of: • 0 h/week (0) • <1 h/week (2.3) • 1–2 h/week (6.8) • 3–4 h/week (15.8) • 5–6 h/week (20.3) • ≥7 h/week (33.8)	RR for T2D relative to most inactive group: 1.00 0.90 (0.82, 0.99) 0.77 (0.69, 0.85) 0.53 (0.45, 0.63) 0.49 (0.38, 0.64) 0.43 (0.31, 0.59)	Age, time period, family history of diabetes, years of education, family income, marital status, cigarette use, alcohol use, energy intake, coffee consumption, television watching, walking
Siegel et al, 2009 [60]	USA; Physicians Health Study	20,757	M	40–84	23.1	8.8 (1836/20,757)	Weekly frequency of vigorous exercise ‘enough to work up a sweat’	Weekly vigorous exercise in number of times/week: • rarely/never (0) • 1–3/month (1.7) • once/week (3.4) • 2–4/week (10.1) • ≥5 times/week (20.3)	RR for T2D relative to most inactive group: 1.00 0.84 (0.72, 0.98) 0.78 (0.68, 0.91) 0.63 (0.55, 0.73) 0.49 (0.41, 0.59) BMI-adjusted RR for T2D relative to most inactive group: 1.00 0.84 (0.72, 0.98) 0.81 (0.70, 0.93) 0.69 (0.61, 0.79) 0.58 (0.48, 0.69)	Age, alcohol use, smoking, history of high cholesterol, history of hypertension (+ BMI in the BMI-adjusted model)
Demakakos et al, 2010 [71]	UK; English Longitudinal Study of Ageing (ELSA)	7466	M/F	62.9–68.3	3.8	3.5 (258/7466)	Frequency/week	Frequency of each vigorous, moderate and low intensity PA: • >1/week • 1/week • 1–3/month • Hardly ever/never Combined to a derived summary three-category index: • physical inactivity (0) • low-intensity but not vigorous/moderate-intensity physical activity at least once a week (3.4) • MVPA or VPA at least once a week (7.0)	HR for T2D relative to most inactive group: 1.00 0.76 (0.51, 1.13) 0.49 (0.33, 0.71) BMI-adjusted HR for T2D relative to most inactive group: 1.00 0.83 (0.56, 1.23) 0.57 (0.39, 0.84)	Age, age-squared, sex, marital status, educational attainment, total household wealth (+ BMI in the BMI-adjusted model)
Ekelund et al, 2012 [39]	Denmark, France, Germany, Italy, Spain, Sweden, UK and the Netherlands; EPIC-InterAct (I)	EPIC total cohort 340,234; InterAct subcohort 15,934; men 6009	M/F; M/F; M	51.4–55.4 (mean)	12.3	3.6 (12,403/340,234); 4.9 (778/15,934); 6.5 (391/6009)	Physical activity index (including OPA)	Four category index which incorporates OPA and LTPA: • ‘inactive’ = sedentary job and no LTPA (0) • ‘moderately inactive’ = sedentary job with 0.5 h LTPA/day or standing job with no LTPA (10.0) • ‘moderately active’ = sedentary job with 0.5–1 h LTPA/day or standing job with 0.5 h LTPA/day or physical job with no LTPA (20.0) • ‘active’ = sedentary job with >1 h LTPA or standing job with 0.5 h LTPA or physical job with some LTPA or heavy manual job (33.4)	HR for T2D relative to most inactive group: 1.00 0.89 (0.78, 1.01) 0.73 (0.64, 0.85) 0.69 (0.60, 0.80)	Education, smoking status, alcohol consumption, energy intake, BMI
Ekelund et al, 2012 [39]	Denmark, France, Germany, Italy, Spain, Sweden, UK and the Netherlands; EPIC-InterAct (II)	EPIC total cohort 340,234; InterAct subcohort 15,934; women 9925	M/F; M/F; F	51.4–55.4 (mean)	12.3	3.6 (12,403/340,234); 4.9(778/15,934); 4(397/9925)	Physical activity index (including OPA)	Four category index which incorporates OPA and LTPA: • ‘inactive’ = sedentary job and no LTPA (0) • ‘moderately inactive’ = sedentary job with 0.5 h LTPA/day or standing job with no LTPA (10.0) • ‘moderately active’ = sedentary job with 0.5–1 h LTPA/day or standing job with 0.5 h LTPA/day or physical job with no LTPA (20.0) • ‘active’ = sedentary job with >1 h LTPA or standing job with 0.5 h LTPA or physical job with some LTPA or heavy manual job (33.4)	HR for T2D relative to most inactive group: 1.00 0.93 (0.89, 0.98) 0.89 (0.78, 1.01) 0.79 (0.68, 0.91)	Education, smoking status, alcohol consumption, energy intake, BMI
Grøntved et al, 2012 [61]	USA; Health Professionals Follow-up Study	32,002	M	44–79	18	7.1 (2278/32,002)	Aerobic exercise min/week	Total time spent on aerobic exercise of at least moderate intensity (≥3 METs); participants grouped into four categories: • none (0) • 1–59 min (2.0) • 60–149 min (7.3) • ≥150 min (27.0)	RR for T2D relative to most inactive group: 1.00 0.93 (0.81, 1.06) 0.61 (0.60, 0.80) 0.55 (0.42, 0.55) BMI-adjusted RR for T2D relative to most inactive group: 1.00 1.00 (0.88, 1.15) 0.80 (0.69, 0.92) 0.61 (0.53, 0.70)	Age, smoking, alcohol consumption, coffee intake, race, family history of diabetes, total energy, trans fat, polyunsaturated fat to saturated fat ratio, cereal fibre, wholegrain, and glycaemic load, weight, physical activity of at least moderate intensity, TV viewing (+ BMI in the BMI-adjusted model)
Lee et al, 2012 [67]	South Korea; National Health Insurance Corporation Study	675,496	M	39.4	7.5	7.9 (52,995/675,496)	Frequency and duration of LTPA that ‘causes sweating’	Physical activity volume was calculated and participants were classified into four categories: • ‘inactive’ (0 min/week) (0) • ‘low’ (1–149 min/week) (5.6) • ‘medium’ (150–299 min/week) (16.9) • ‘high’ (≥300 min/week) (28.1)	HR for T2D relative to most inactive group: 1.00 0.98 (0.96, 0.99) 0.94 (0.91, 0.96) 0.94 (0.91, 0.97) BMI-adjusted HR for T2D relative to most inactive group: 1.00 0.95 (0.93, 0.97) 0.90 (0.87, 0.93) 0.91 (0.89, 0.94)	Age, smoking status, alcohol intake, hypertension, parental diabetes, baseline glucose (+ BMI in the BMI-adjusted model)
Steinbrecher et al, 2012 [38]	USA; The Multiethnic Cohort (I)	35,976 (men)	M	45–75	14	12.6 (4527/35,927)	H/week of strenuous sport, vigorous work or moderate activity	Physical activity frequency for strenuous sport was collapsed into four categories: • never (0) • 0.5–1 h/week (3.4) • 2–3 h/week (11.3) • >4 h/week (20.3)	HR for T2D relative to most inactive group: 1.00 0.94 (0.87, 1.02) 0.85 (0.77, 0.94) 0.80 (0.72, 0.88)	Age, ethnicity, education, BMI
Steinbrecher et al, 2012 [38]	USA; The Multiethnic Cohort (II)	38,937 (women)	F	45–75	14	10.4 (4034/38,937)	H/week of strenuous sport, vigorous work or moderate activity	Physical activity frequency for strenuous sport was collapsed into four categories: • never (0) • 0.5–1 h/week (3.4) • 2–3 h/week (11.3) • >4 h/week (20.3)	HR for T2D relative to most inactive group: 1.00 1.00 (0.91, 1.09) 0.85 (0.75, 0.96) 0.67 (0.57, 0.79)	Age, ethnicity, education, BMI
Shi et al, 2013 [51]	China; Shanghai Men’s Health Study^d^	51,464	M	54.1	5.4	2.5 (1304/51,464)	Appraisal of LTPA, DPA and CPA Participants had to indicate whether they had undertaken any LTPA ≥1/week over the preceding 5 years	LTPA volume was reported as four categories of MET h/week/year • none (0) • low (<1.2) (4.2) • medium (1.2–3) (14.7) • high (≥3) (27.3)	HR for T2D relative to most inactive group: 1.00 0.79 (0.65, 0.96) 0.87 (0.72, 1.04) 0.87 (0.75, 1.07) BMI-adjusted HR for T2D relative to most inactive group: 1.00 0.80 (0.65, 0.97) 0.89 (0.74, 1.07) 0.91 (0.76, 1.08)	Age, energy intake, smoking, alcohol consumption, education level, occupation, income level, hypertension, family history of diabetes (+ BMI and WHR in further adjusted model)
Fan et al, 2014 [59]	China; China Multicenter Collaborative Study of Cardiovascular Epidemiology (China MUCA) and China Cardiovascular Health Study	6348	M/F	49.2	7.9	7.5 (478/6348)	Physical activity level (PAL) = method to estimate total daily energy expenditure (80)	Average h/day spent in vigorous activity (e.g. jogging), moderate activity (e.g. yard work), light activity (e.g. office work), sedentary activity (e.g. TV) and periods of reclining during the previous 12 months Four PAL categories: • sedentary (PAL 1.00–1.39) (136.2) • low active (PAL 1.40–1.59) (173.0) • active (PAL 1.60–1.89) (202.9) • very active (PAL >1.89) (238.6)	HR for T2D relative to most inactive group: 1.00 0.92 (0.69, 1.22) 0.70 (0.52, 0.93) 0.55 (0.42, 0.73) BMI-adjusted HR for T2D relative to most inactive group: 1.00 0.82 (0.62, 1.09) 0.63 (0.47, 0.83) 0.47 (0.36, 0.61)	Age, sex, geographic region, educational level, cigarette smoking, alcohol consumption, family history of diabetes (+ BMI in the BMI adjusted model)
Grøntved et al, 2014 [35]	USA; Nurses’ Health Study (2000–2008) (I)	51,642	F	53–81	8	4.2 (2158/51,642)	MVPA min/week	MVPA defined as brisk walking, jogging, running, bicycling, tennis, swimming, other aerobic exercise, other vigorous exercise and stair climbing (>3 METs) and categorised into quintiles according to average min/week: • none (0) • 1–29 (1.1) • 30–59 (3.4) • 60–150 (7.9) • >150 (14.6)	RR for T2D relative to most inactive group: 1.00 0.84 (0.73, 0.97) 0.76 (0.66, 0.88) 0.68 (0.60, 0.77) 0.48 (0.42, 0.54) BMI-adjusted RR for T2D relative to most inactive group: 1.00 0.94 (0.81, 1.09) 0.88 (0.76, 1.02) 0.85 (0.74, 0.96) 0.66 (0.58, 0.75)	Race, alcohol, weight training, coffee intake, smoking, postmenopausal hormone use, oral contraceptive use, menopausal status, family history of diabetes, total calorie intake, saturated to polyunsaturated fat ratio, trans fat, cereal fibre, wholegrains, glycaemic load (+ BMI in the BMI-adjusted model)
Grøntved et al, 2014 [35]	USA; Nurses’ Health Study II (2001–2009) (II)	47,674	F	36–55	8	2.8 (1333/47,674)	MVPA min/week	MVPA defined as brisk walking, jogging, running, bicycling, tennis, swimming, other aerobic exercise, other vigorous exercise, and stair climbing (>3 METs) and categorised into quintiles according to average min/week: • none (0) • 1–29 (1.1) • 30–59 (3.4) • 60–50 (7.9) • >150 (14.6)	RR for T2D relative to most inactive group: 1.00 0.80 (0.67, 0.95) 0.68 (0.57, 0.82) 0.63 (0.54, 0.74) 0.42 (0.36, 0.50) BMI-adjusted RR for T2D relative to most inactive group: 1.00 0.94 (0.79, 1.13) 0.83 (0.69, 1.00) 0.86 (0.73, 1.01) 0.70 (0.59, 0.83)	Race, alcohol, weight training, coffee, smoking, post-menopausal hormone use, oral contraceptive use, menopausal status, family history of diabetes, total calorie intake, saturated to polyunsaturated fat ratio, trans fat, cereal fibre, wholegrains, glycaemic load (+ BMI in the BMI-adjusted model)
Ding et al, 2015 [42]	Australia; 45 and Up study	54,997	M/F	≥45	3.4	1.6 (888/54,997)	PA calculated as the sum of time spent in walking, MVPA and VPA (weighted by a factor of two), in the previous week	Total min MVPA/week: • <150 min (5.6) • 150–<300 min (16.9) • ≥300 min (28.1)	OR for T2D relative to most inactive group: 1.00 0.72 (0.56, 0.94) 0.71 (0.85, 0.97)	Age, sex, BMI, SES, health status, BP, blood cholesterol, weight, family history of T2D/heart disease, smoking, alcohol, sitting time, sleep, fruit and vegetable intake, psychological distress

This is an abridged version of ESM Table 4, which includes details of the method of PA assessment and additional comments

^a^Doses were assigned from descriptions identified within the individual studies or from correspondence with study authors. Full details of MET h dose assignment are listed in ESM Table 2, together with the MMET h/week calculations (see ESM)

^b^Studies updated with further follow-up data obtained from the authors

^c^Total cohort

^d^Studies included in the sensitivity analysis using variance-weighted least squares regression analysis

(I)/(II) indicate subcohorts with independently reported risk estimates for T2D within the same publication

CCCC: Chin-Shan community cardiovascular cohort study; China MUCA: China Multicenter Collaborative Study of Cardiovascular Epidemiology; CPA, commuting physical activity; CVD, cardiovascular disease; DPA: daily living physical activity; EE, energy expenditure; ELSA: English longitudinal study of ageing; EPIC-InterAct: European Prospective Investigation into Cancer and Nutrition-InterAct; F, Female; FPG, fasting plasma glucose; M, Male; MEC, Multiethnic cohort; MONICA/KORA: Monitoring Trends and Determinants on Cardiovascular Diseases/Cooperative Research in the Region of Augsburg Cohort Study; NHS, Nurses’ Health Study; OPA, occupational physical activity; SES, socioeconomic status

Age was the only variable for which all cohorts had adjusted their findings, with adjustment for other confounders varying considerably. Four cohorts [14, 36, 58, 64] did not adjust for BMI, a key variable believed to mediate the effect of PA on type 2 diabetes. Overall, inverse associations between PA and incident type 2 diabetes were observed for all identified cohorts.

### Linear association between PA and incidence of type 2 diabetes

Study-specific linear RRs (95% CI) for 10 MET h/week increments of PA sorted by PA domain and publication year, are shown in Fig. 1.

The mean pooled risk reduction for type 2 diabetes was 13% (95% CI 11%, 16%) per 10 MET h/week increment of PA, albeit observed in the presence of high heterogeneity (*I* ^2^ 93.5%, *p*_Het_ < 0.001). Consecutive removal of single studies indicated no significant impact of any one study on the overall heterogeneity in the model (*I* ^2^ 88.3–92.3%, *p*_Het_ < 0.001). Likewise, restriction to studies rated as high quality did not substantially influence model heterogeneity (*I* ^2^ 82%, *p*_Het_ < 0.001, *n* = 17).

Risk reductions for type 2 diabetes were considerably more pronounced for LTPA compared with the benefits estimated for total PA. Each 10 MET h/week increment of LTPA reduced type 2 diabetes risk by 17% (95% CI 13%, 21%) compared with 5% (95% CI 2%, 7%) for each 10 MET h/week increment of total PA. Benefits from VPA integrated over time to MET h/week were much larger, with a decrease in risk of type 2 diabetes of 56% (95% CI 16%, 77%) per 10 MET h/week increment.

The effects appeared to be more pronounced in women with a pooled RR of 0.83 (95% CI 0.77, 0.90, *I* ^2^ 89.5%, *p*_Het_ < 0.001, *n* = 10 observations) compared with a pooled RR for men of 0.89 (95% CI 0.86, 0.93, *I* ^2^ 95.3%, *p*_Het_ < 0.001, *n* = 13 observations) per 10 MET h/week. Studies conducted in Asia on average observed less benefit, with a mean RR of 0.97 (95% CI 0.95, 0.98, *I* ^2^ 65.2%, *p*_Het_ < 0.001, *n* = 6 observations) per 10 MET h/week when compared to the USA (0.85 [95% CI 0.79, 0.91, *I* ^2^ 96.6%, *p*_Het_ < 0.001, *n* = 13]) or Europe (0.83 [95% CI 0.77, 0.89, *I* ^2^ 80.6%, *p*_Het_ < 0.001, *n* = 11 observations]). The two studies in Australia reported, on average, the highest benefit (0.81 [95% CI 0.65, 1.01, *I* ^2^ 77.1%, *p*_Het_ < 0.001]; see Table 2).

**Table 2:** Relative risk estimates for type 2 diabetes per 10 MET h/week of physical activity, stratified by study design and population characteristics

Characteristic	RR per 10 MET h/week	95% CI	*I* ^2^ (%)	*p*_Het_	Independent observations (*n*)	Incident cases of type 2 diabetes
Degree of adjustment						
Overall pooled estimates	0.87	0.84, 0.89	93.5	<0.001	32	84,144
BMI unadjusted	0.81	0.77, 0.84	96.8	<0.001	21	70,251
BMI adjusted	0.87	0.84, 0.90	92.6	<0.001	27	80,505
Sex						
M	0.89	0.86, 0.93	95.3	<0.001	13	11,282
F	0.83	0.77, 0.90	89.5	<0.001	10	16,317
M/F	0.84	0.78, 0.91	86.9	<0.001	9	56,545
Follow up						
< 10 years	0.92	0.90, 0.95	86.1	<0.001	16	69,849
> 10 years	0.84	0.80, 0.89	90.6	<0.001	16	14,295
Location						
Europe	0.83	0.77, 0.89	80.6	<0.001	11	55,440
N America	0.85	0.79, 0.91	96.6	<0.001	13	17,074
Asia	0.97	0.95, 0.98	65.2	0.01	6	10,518
Australia	0.81	0.65, 1.01	77.1	0.04	2	1112
Study quality						
High (≥7 stars)	0.93	0.90, 0.95	82.0	<0.001	17	17,131
Medium to low	0.81	0.75, 0.88	96.2	<0.001	15	67,013
BMI						
< 30 kg/m^2^	0.75	0.65, 0.95	63.1	0.01	4	907
> 30 kg/m^2^	0.88	0.80, 0.96	0.00	<0.001	3	1155
PA intensity						
VPA only	0.44	0.23, 0.84	0.00	0.01	2	118
PA domain						
Total PA	0.95	0.93, 0.98	85.6	<0.001	5	1825^a^
LTPA	0.83	0.79, 0.87	92.7	<0.001	27	82,319

Pooled RRs based on 28 cohorts (32 independent observations) with a total population sample size of *n* = 1,261,991 and a total of 84,134 incident cases of type 2 diabetes

MVPA is defined as an average intensity of 4.5 MET/h. VPA defined as an average intensity of 8 MET/h

^a^Total PA incident cases of T2D are *n* = 13,444 if observations from the entire EPIC cohort [39] are included

Adjustment for BMI appeared to attenuate the pooled protective effect size by around one-third, from 0.81 (95% CI 0.77, 0.84, *I* ^2^ 96.8%, *p*_Het_ < 0.001, *n* = 21 observations) to 0.87 (95% CI 0.84, 0.90, *I* ^2^ 92.6%, *p*_Het_ < 0.001, *n* = 27 observations). Stratification by participant BMI suggested the protective effect of activity was more pronounced in those with BMI < 30 kg/m^2^, with an observed mean RR of 0.75 (95% CI, 0.65, 0.95, *I* ^2^ 63.1%, *p*_Het_ = 0.01, *n* = 4 observations) vs 0.88 (95% CI 0.80, 0.96, *I* ^2^ 0.00, *p*_Het_ < 0.001, *n* = 3 observations) for obese individuals. Inspection of funnel plots and Egger’s test for asymmetry (*p* < 0.001) did not indicate the presence of publication bias or small studies effect (ESM Fig. 3).

### Non-linear dose–response analysis

**Fig. 2:**
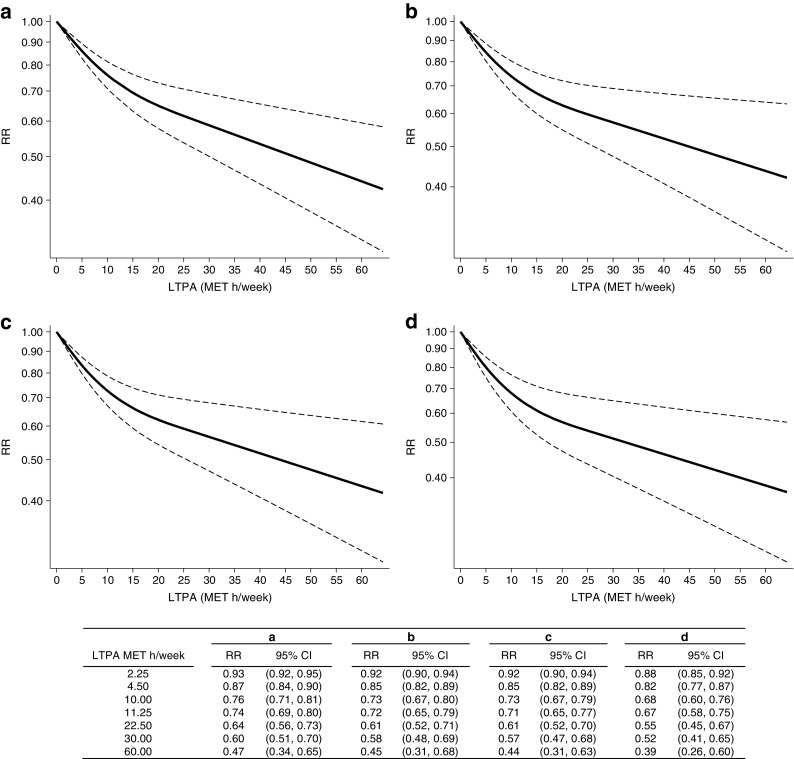
(**a**–**d**) Dose–response association between LTPA and incidence of type 2 diabetes modelled using restricted cubic splines and comparison of predicted RR point estimates for type 2 diabetes using different dose-assignment assumptions. LTPA converted to MET h/week with results pooled in a two-stage random-effects model. RRs were derived from a common lowest PA category within each study. Listed exposure levels were chosen to represent meaningful and easy to interpret PA volumes equivalent to the following: 30 min of MVPA; 1 h MVPA; rounded value to allow for comparison with GLS PA exposure increment; 150 min PA/current recommended guidelines; double the recommended guidelines and two high PA exposure levels investigating the risk reductions at the higher end of the LTPA spectrum. The bold lines indicate the pooled restricted cubic spline model and the black dashed line indicates the 95% CIs of the pooled curve. Duration assumption was necessary in nine out of 27 observations, applied as 45 min/session in scenarios (**a**) and (**c**), and 30 min/session in scenarios (**b**) and (**d**). Intensity assumption was necessary in 15 out of 27 observations, applied as low-intensity PA (LPA) = 3 MET, MVPA = 4.5 MET and VPA = 8 MET in scenarios (**a**) and (**b**), and LPA = 2 MET, MVPA = 3.5 MET and VPA = 7 MET in scenarios (**c**) and (**d**)

In total, data from 23 cohorts were included in the restricted cubic spline analysis and the ensuing pooling in a two-stage multivariate dose–response model. A significant non-linear dose–response is shown in Fig. 2a (*p*_Non-linearity_ < 0.001), with greater risk reduction at moderate exposures compared with higher ones.

Results from the cubic spline model suggest that individuals who accumulate 11.25 MET h/week (equivalent to meeting the recommended guidelines of 150 min/week of activity at 4.5 MET) have a reduced risk of developing type 2 diabetes equal to 26% (95% CI 20%, 31%) relative to completely inactive individuals.

We found no indication of a substantial threshold effect or plateau for the obtained benefit across increasing levels of PA. Being active at a level corresponding to double that of the recommended minimal PA (22.5 MET h/week) was associated with a reduced risk of type 2 diabetes of 36% (95% CI 27%, 46%) with further reductions at higher doses (60 MET h/week, risk reduction of 53%), in the cubic spline model.

For 8.75 MMET h/week (equivalent to 11.25 MET h/week at a mean gross intensity of 4.5 MET) the pooled RR for type 2 diabetes was 0.74 (95% CI 0.69, 0.80), with risk being 0.64 (95% CI 0.56, 0.73) for those doing twice as much. Point risk estimates of the pooled dose–response relation for LTPA (in MET h/week) and type 2 diabetes are tabulated in Fig. 2 (also available online as an interactive version at http://epiweb.mrc-epid.cam.ac.uk/meta-analyses/pa/diabetes/).

Sensitivity analyses were run to assess the effect of assumptions regarding duration or intensity of the PA exposure used in the LTPA dose assignment procedure for those studies where this information was not directly available; see Fig. 2 b-d and ESM Fig. 4. The shape of the dose–response curve was similar under these different assumptions. Benefits were larger for a given exposure if duration and intensity were assumed to be smaller in the original studies where these assumptions were needed. Furthermore, we repeated the final cubic spline model including variance-weighted linear dose–response gradients of the two identified studies that could not be used in the main model because of incomplete data. The impact of excluding these studies was minimal on the overall final result, with a risk reduction of 24% (95% CI 19%, 29%) at 11.25 MET h/week in this more inclusive model (ESM Table 3 and ESM Fig. 4).

## Discussion

Our results from a comprehensive literature search identifying relevant longitudinal studies indicate an inverse association between PA and incidence of type 2 diabetes, which was consistently observed across the identified cohorts. Using the restricted cubic splines model, accumulating an activity volume which is commensurate with adherence to the current public health recommendations of 150 min of MVPA per week compared with sedentary individuals was associated with a reduction in the risk of type 2 diabetes by 26% (95% CI 20%, 31%) in the general population.

Our results suggest that the benefits of higher activity levels extend considerably beyond the minimum recommendations. Using the restricted cubic spline model we found that a doubling of activity volume from 11.25 MET h/week to 22.5 MET h/week would further reduce the risk of type 2 diabetes by 10% to a total risk reduction of 36% compared with being inactive. For an intensity of 4.5 MET, our results were very similar under the MMET analysis. However, a greater benefit would be gained from using MMETs for more intensive activity, whereas less intensive activity would gain smaller benefits.

Central to any dose–response analysis for assessing PA in relation to health is the issue of uncertainty in the way by which PA was assessed in free-living individuals. Self-reported PA generally correlates significantly but weakly with objective methods of PA ascertainment, with approximately 10% shared variance [60]. A further crucial issue which may have affected our findings is the substantial heterogeneity in the measurement and reporting of PA behaviour, resulting from questionnaires ascertaining different domains, timeframes and/or units of PA. Methods of outcome assessment were also not consistent across the identified cohorts and it is possible that diagnostic bias may have distorted the results of some of the studies because of differences in diabetes detection accuracy.

When interpreting the findings, the fact that most studies were primarily conducted in samples of well-educated white populations in high-income countries must be taken into account. In the context of type 2 diabetes, earlier studies have found that dose–response curves may be different for Asian Indians who may require more PA to be protected from their relatively higher susceptibility to develop type 2 diabetes [72, 73].

A potential strength of our present analyses is the expression of PA exposure dose in MMET h/week rather than just MET h/week. There is a fine distinction between these two measures; an individual expending 3 METs on a given activity is using double the activity-related energy above rest than an individual performing an activity at 2 METs. By setting the starting point of the PA volume at 0 MMET h/week, better mathematical properties (proportionality) of the exposure variable are taken into account, allowing different intensities of activity to be more fairly equated, both within and across individuals and populations. This calculation gives a relatively higher weighting to time spent in more vigorous activity compared with classic METs. This means that doing more intensive activity would equate to a relatively larger dose in the MMET model than under the MET model. For example, 2.5 h/week of MVPA at 4.5 MET (equal to 11.25 MET h/week) is volume equivalent to 1.41 h of 8 MET of intense activity, while 2.5 h/week of MVPA at 3.5 MMET (equal to 8.75 MMET h/week) is volume equivalent to 1.25 h of 7 MMET of intense activity. Results for MVPA were similar, but benefits were larger for more intense PA.

Most cohorts were not designed to specifically investigate PA and the resulting paucity of comprehensive data on all PA behaviours may have hindered our analysis. We used aggregated exposure measures across a range of reported activities from each study, which relied on the originally assigned intensity values for each activity by the primary study analysis alongside aggregated durations, however it is likely that more accurate MMET h estimates could be calculated with access to individual-level raw PA data. Nevertheless, expressing PA in marginal MET units is a promising method to account for activities of differing intensity and would be aided by better reporting of intensity and duration characteristics for each exposure group.

As a restricted cubic spline regression model was used to study the shape of the dose–response relationship, we were able to improve precision as to how the association between PA and incident type 2 diabetes varies at different exposure levels [49].

An earlier systematic review [25] also conducted dose–response meta-analyses for PA and type 2 diabetes. However, this review achieved far less data harmonisation than in our paper. Aune et al report results separately for MET h/week (five studies), hours per week (ten studies) and energy expenditure (four studies). They found a larger benefit (based on an assumption of moderate intensity activity) and a more linear dose–response curve using the time-based measure compared with the MET h measure. Our results, which are derived from 23 studies, suggest considerably larger benefits for the same PA exposure level, e.g. RR of 0.65 vs RR of 0.76 at 20 MET h/week. Given that our more extensive approach to harmonisation requires more assumptions it is encouraging that our sensitivity analysis found relatively small differences in the size of the effects, and little difference in the shape of the dose–response curve.

Previous research into PA and other health outcomes has often provided evidence in favour of a strongly curvilinear dose–response relationship [20–23, 74]. This curvilinear association has been the basis for further health impact modelling studies [75] and, as such is used to estimate how much gain there would be in population health from different PA interventions or scenarios. Uncertainty about the dose–response shape has been found to contribute substantially to uncertainty about the final results of partaking in PA for disease prevention. Our results indicate that for type 2 diabetes prevention, while probably curvilinear over a much wider exposure range, the relationship is much closer to linearity than that found previously for all-cause mortality or ischaemic heart disease [21]. Our effect estimates are likely to be conservative, given the diluting impact that exposure measurement error stemming from a single self-report measure of activity will have on the observed associations. Even so, our results suggest a major potential for PA to slow down or reverse the global increase in type 2 diabetes prevalence and should prove useful for health impact modelling, which frequently forms part of the evidence base for policy decisions (e.g. WebTAG for transport [76]).

Increasingly, PA research is incorporating the use of objective data, e.g. UK Biobank has recently collected accelerometry data in 100,000 individuals who are also followed up over time to link this data with health outcomes. However, before such studies accrue enough major clinical events to examine prospective relationships, self-report data may be calibrated against objective measures to enhance translation of findings based on self-report into public health action [77].

Given the non-linear nature of the dose–response curve between LTPA and type 2 diabetes, the effects of LTPA are likely to depend on the exposure to non-leisure activity. Our finding of a smaller effect for total PA is unexpected but was based on a much smaller evidence base and may reflect differences in measurement properties between domains. Assuming, however, that the non-linear relationship holds across all domains, the marginal effect of LTPA will be greater in a population that is less active in other domains and vice versa. One way to address this would be to conduct a meta-analysis of LTPA by level of non-leisure PA, e.g. occupational grouping.

The results from this dose–response meta-analysis provide evidence in support of the clinically meaningful role of PA in the primary prevention of type 2 diabetes in the general population. We highlight the necessity for progress in PA measurement and reporting of PA of different intensities and duration in cohort studies. Additionally, we recommend investigations to consider the dose–response relationship of PA and type 2 diabetes prevention in more ethnically diverse population groups.

Overall, we found the dose–response curve for PA and incident type 2 diabetes is curvilinear. Our study suggests that notable health benefits of PA can be realised even at relatively low levels of PA but also that considerable additional decreases in risk for type 2 diabetes are afforded when substantially exceeding the current PA guidelines.

Our meta-analysis supports the generally accepted notion of a graded association between PA and health maintenance [78, 79]. It favours a *‘*some is good but more is better’ guideline, in which specific targets are mainly used for a psychological effect. There is no clear cut-off at which benefits are not achieved and health protection increases at activity levels well beyond current recommendations. Enabling cultures and built environments to increase PA at the population-wide level may prevent substantial personal suffering and economic burden. Given the current obesity and diabetes epidemic, the utility of such a strategy may reach beyond any present-day approaches to improve population health.

## Electronic supplementary material

Below is the link to the electronic supplementary material.

ESM 1 (PDF 1.04 mb)

## Abbreviations

GLS: Generalised least-squares
LPA: Low-intensity physical activity
LTPA: Leisure-time physical activity
MET: Metabolic equivalent of task
MMET: Marginal metabolic equivalent of task
MVPA: Moderate to vigorous physical activity
NOS: Newcastle Ottawa Scale
PA: Physical activity
VPA: Vigorous physical activity
