# Supplementary material for: Physical activity and incident type 2 diabetes mellitus: a systematic review and dose–response meta-analysis of prospective cohort studies
Source: Diabetologia. 2016 Oct 17;59(12):2527–45. doi: 10.1007/s00125-016-4079-0 (PMC6207340; doi:10.1007/s00125-016-4079-0)
Supplement: Supplementary file 1 — (PDF 1.04 mb) [file 125_2016_4079_MOESM1_ESM.pdf]

Electronic Supplementary Material

Physical activity and incident type 2 diabetes mellitus: A systematic review and dose-response meta-analysis of prospective cohort studies

Andrea D Smith, Alessio Crippa, James Woodcock, Søren Brage

**Table of Content**	**Page number ESM**
**ESM Table 1** Newcastle-Ottawa quality scores of 28 identified prospective cohort studies retrieved on the association of physical activity and type 2 diabetes	2
**ESM Table 2** Summary of MET h/week and MMET h/week dose assignment calculations for the prospective cohort studies included in the dose-response meta-analysis	6
**ESM Table 3** Summary of MET h/week and MMET h/week sensitivity analysis dose assignment calculations for the prospective cohort studies included in the dose-response meta-analysis	15
**ESM Table 4** Summary of the characteristics of 28 prospective cohort studies that investigate the association between levels of PA and incident type 2 diabetes, identified in the systematic literature search	20
**ESM Figure 1** Systematic literature review search terms and strategy	31
**ESM Figure 2** PRISMA flow chart	32
**ESM Figure 3** Funnel plot for the linear association of the RR for diabetes type 2 against the standard error of the RR per 10 MET h/week increment of physical activity	33
**ESM Figure 4** Comparison of the dose-response association and predicted RR point-estimates between LTPA and incidence of type 2 diabetes between the model using restricted cubic splines and the restricted cubic spline model including linear trend estimates of studies not eligible for flexible modelling	34

ESM Table 1 Newcastle-Ottawa quality scores of 28 identified prospective cohort studies retrieved on the association of physical activity and type 2 diabetes

	Study Selection				Comparability of cohorts		Outcome			Total Score
	Representativeness of the exposed cohort	Selection of the non-exposed cohort	Ascertainment of exposure	Demonstration that outcome of interest was not present at start of study	BMI	PA intensity	Assessment of outcome	Length of follow up	Adequacy of follow up	
**Physicians Health Study** (1)	C	A*	C	C	A*	B	C	A*	B*	4
**Nurses' Health Study** (2)	C	A*	C	C	A*	A*	C	A*	B*	5
**British Regional Heart Study** (3)	B*	A*	C	B*	A*	A*	B*	A*	A*	8
**Women's Health Study** (4)	C	A*	C	C	A*	A*	C	A*	B*	5
**Eastern and South-Western Finnish adults** (5)	A*	A*	C	A*	A*	A*	B*	A*	B*	8
**Iowa Women's Health Study** (6)	B*	A*	C	C	A*	A*	C	A*	B*	6
**University of Pennsylvania Alumni** (7)	C	A*	C	C	B	A*	C	A*	C	3
**North-Eastern Finnish Adult cohort** (8)	A*	A*	C	C	B	A*	C	A*	C	4
**MONICA/KORA Augsburg Cohort Study** (9)	A*	A*	B*	C	A*	B	B*	A*	B*	8

	Study Selection				Comparability of cohorts		Outcome			Total Score
	**Representativeness of the exposed cohort**	**Selection of the non-exposed cohort**	**Ascertainment of exposure**	**Demonstration that outcome of interest was not present at start of study**	**BMI**	**PA intensity**	**Assessment of outcome**	**Length of follow up**	**Adequacy of follow up**	
**Health Professional's Follow-up Study** (10)	C	A*	C	C	A*	A*	C	A*	B*	5
**Osaka Health Survey** (11)	C	A*	C	A*	A*	B*	A*	A*	B*	7
**Shanghai Women's Health Study** (12)	B*	A*	B*	A*	A*	A*	C	A*	B*	8
**Kuopio Ischaemic Heart Disease Risk Factor Study**(13)	B*	A*	C	A*	A*	A*	A*	A*	B*	8
**The Pitt County Study**(14)	C	A*	C	A*	A*	A*	A*	A*	C	6
**The Strong Heart Study**(15)	C	A*	C	A*	A*	A*	A*	A*	B*	7
**English Longitudinal Study of Ageing** (16)	B*	A*	C	C	A*	A*	C	A*	B*	6
**National Health Insurance Corporation Study**(17)	B*	A*	C	A*	A*	B	A*	A*	B*	7
**The Multiethnic Cohort** (18)	A*	A*	C	B*	A*	A*	B*	A*	B*	8
**Chin-Shan community cardiovascular cohort study (CCCC)** (19)	A*	A*	C	A*	A*	B	A*/B*	A*	B*	7

	Study Selection				Comparability of cohorts		Outcome			Total Score
	Representativeness of the exposed cohort	Selection of the non-exposed cohort	Ascertainment of exposure	Demonstration that outcome of interest was not present at start of study	BMI	PA intensity	Assessment of outcome	Length of follow up	Adequacy of follow up	
**Black Women's Health Study** (20)	C	A*	C	C	B	A*	B*	A*	C	4
**The Australian Diabetes, Obesity and Lifestyle Study** (21)	A*	A*	B*	A*	A*	A*	A*	A*	B*	9
**The Nord-Trøndelag Health Survey** (22)	A*	A*	C	C	A*	B	A*	A*	B*	6
**45 and Up study** (23)	A*	A*	C	C	A*	A*	C	A*	C	5
**Honolulu Heart Program** (24)	C	A*	C	A*	B	A*	C	A*	B*	5
**InterAct Consortium**(25)	B*	A*	C	A*	A*	A*	A*	A*	B*	8
**China MUCA and China Cardiovascular Health Study** (26)	A*	A*	C	A*	A*	A*	A*	A*	B*	8
**Japanese Office Workers**(27)	C	A*	C	A*	A*	A*	A*	A*	B*	7

Key to NOS star allocation

Selection
1) Representativeness of the exposed cohort
A*= truly representative of the general population
B* = somewhat representative of the general population
C = selected group of users e.g. nurses, volunteers
2) Selection of the non-exposed cohort
A* = drawn from the same community as the exposed cohort
B = drawn from a different source
3) Ascertainment of exposure
B* = Face-to-face structured interview
C = Self-administered questionnaire
4) Demonstration that outcome of interest was not present at start of study
A* = Clinical assessment
B* = Medical records, medication status of the patient
C = Self-report
D = No demonstration

Comparability
1) Comparability of cohorts on the basis of the design or analysis
A* = study adjusts for BMI
B = study doesn't adjust for BMI
2) Comparability of cohorts on the basis of the design or analysis
A* = study assesses varying levels of physical activity intensity (not only duration)
B = study doesn't asses varying levels of physical activity intensity

Outcome
1) Assessment of outcome
A* = independent blind clinical assessment
B* = record linkage
C = self-report
2) Was follow-up long enough for outcomes to occur
A* = Yes (> 2 years)
B = No
3) Adequacy of follow up of cohorts
A*= complete follow up (>= 99%)
B * = subjects lost to follow up unlikely to introduce bias > 80% subjects followed up or description of those lost suggests unlikely to introduce bias
C = follow up rate < 80% and no description of those lost
D = no statement on follow up

ESM Table 2 Summary of MET h/week and MMET h/week dose assignment calculations for the prospective cohort studies included in the dose-response meta-analysis

Study	Frequency	Duration	Intensity	Assigned MET h/week	MMET h/week	Additional information
**Physicians Health Study** (28)	•0<br>•1<br>•2-4 (mid-point: 3)<br>•>5 (mid-point: 6)	45 min/session	MVPA = 4.5 MET	0<br>3.375<br>10.125<br>20.25	0<br>2.625<br>7.875<br>15.75	
**Nurses' Health Study** (2)		Average minutes/week<br><br>• None<br>• 1–29<br>(mid-point: 15)<br>• 30–59<br>(mid-point: 45)<br>• 60–150<br>(mid-point: 105)<br>• >150<br>(mid-point: 195)	MVPA = 4.5 MET	0<br>1.125<br>3.375<br>7.875<br>14.625	0<br>0.875<br>2.625<br>6.125<br>11.375	
**British Regional Heart Study** (3)	•"Inactive/occasional" (irregular PA)<br>•"Light" (more frequent recreational activities or PA <1/week, or regular walking + recreational activity)<br>•"Moderate" (frequent weekend recreational activities + regular walking, or PA 1x/week)<br>•"Moderately vigorous" (exercise 1/week or frequent cycling + recreational activities or walking or frequent sporting activities)<br>•"Vigorous" (very frequent exercise, or frequent exercise + recreational activities)	45 min/session<br><br>• "Inactive":<br>30 min/week<br>•"Light": At least 1/week = 1x/week<br>• "Moderate": frequent recreational weekend activities and sporting activity 1/week = MVPA 2x/week<br>• "Moderately vigorous"<br>= 150 min/week<br>• "Vigorous" =MVPA 5x/week	MVPA = 4.5 MET	2.5<br>3.375<br>6.75<br>11.25<br>16.875	1.75<br>2.625<br>5.25<br>8.75<br>13.125	

Study	Frequency	Duration	Intensity	Assigned MET h/week	MMET h/week	Additional information
**Nurses' Health Study (1980 - 2010)** (29)		Average weekly hours of MVPA:<br>• <0.5 h (15 min)<br>•0.5–1.9 h (75 min)<br>•2–3.9 h (180 min)<br>•4–6.9 h (330 min)<br>•>7 h (510 min)	MVPA = 4.5 MET	7.8<br>13.1<br>22.4<br>34.3<br>68	6.1<br>10.2<br>17.4<br>26.3<br>53	Updated follow up data and mean MET h durations per exposure category were obtained from the authors to estimate doses for this cohort
**Women's Health Study** (4)			Kcal EE/week categories for LTPA:<br>• 0–199 (mid-point: 99.5)<br>• 200–599 (mid-point: 299.5)<br>• 600–1499 (mid-point: 749.5)<br>• >1500 (mid-point: 1645.5)	1.37<br>4.1<br>10.34<br>22.69	1.04<br>3.2<br>8.04<br>17.65	Conversion from EE/ week to MET h/ week according to:<br>LTPA EE/week/72.5=LTPA MET h/week<br><br>72.5 kg as reported on http://whs.bwh.harvard.edu/participants.html
**Eastern and South-Western Finnish adults** (5)	• "Low" = light levels of OPA & CPA (<30 minutes) & inactive LTPA<br>• "Moderate" = 1 type of LTPA activities/week (= 2 sessions<br>• "High" = 2 or 3 types of LTPA/week (Each 2 x per week) = 4 sessions	45 min/session	MVPA = 4.5 MET	0<br>6.75<br>13.5	0<br>5.25<br>10.5	
**Iowa Women's Health Study** (6)	• Rare or never<br>• 1/ week - few times/month (low)<br>• 2–4/ week (medium)<br>• >4 per week (high)	45 min/session<br>• Rare/ never (= 0 min)<br>• Low (= 45 min/week)<br>• Medium (= 135 min/week)<br>• High (= 225 min/week)	MVPA = 4.5 MET	0<br>3.375<br>10.125<br>16.9	0<br>2.625<br>7.875<br>13.15	

Study	Frequency	Duration	Intensity	Assigned MET h/week	MMET h/week	Additional information
**University of Pennsylvania Alumni** (7)			Weekly total EE for LTPA in 500 kcal increments:<br>•<500 (mid-point: 250)<br>•500 – 999 (mid-point: 750)<br>•1000-1499 (mid-point: 1250)<br>•1500 -1999 (mid-point: 1750)<br>•2000 -2499 (mid-point: 2250)<br>•2500 -2999 (mid-point: 2750)<br>•3000 – 3499 (mid-point: 3250)<br>•≥ 3500 (mid-point: 3750)	3.28<br>9.84<br>16.4<br>22.97<br>29.53<br>36.09<br>42.65<br>49.21	2.55<br>7.65<br>12.76<br>17.87<br>22.97<br>28.07<br>33.17<br>38.27	Conversion from EE/ week to MET h/ week according to the following formula: LTPA EE/week/76.2=LTPA MET h/week<br><br>Average weight of males in the general population: 168 lbs (= 76.2 kg) ((30))
**North-Eastern Finnish Adult cohort** (8) **(I)**			Weekly LTPA EE (kcal) categories for men defined as:<br>•Low: 0–1100 (mid-point: 550)<br>•Moderate: 1101–1900 (mid-point: 1500.5)<br>•High: >1900 (mid-point: 2299)	6.8<br>18.5<br>28.3	5.3<br>14.4<br>22	LTPA EE/week/81.3=LTPA MET h/week<br><br>BMI: 25.1<br>Height: 180 cm<br>Mean weight: 81.3 kg<br><br>Height info: http://www.pisa2006.helsinki.fi/finland/statistics/statistics.htm<br><br>Weight information source: ((31))
**North-Eastern Finnish Adult cohort** (8) **(II)**			Weekly LTPA EE (kcal) categories for women:<br>• Low: 0–900 (mid-point: 450)<br>• Moderate: 901–1500 (mid-point: 1200.5)<br>• High >1500 (mid-point: 1799.5)	6.9<br>18.3<br>25.7	5.4<br>14.2<br>20	LTPA EE/week/65.6=LTPA MET h/week<br><br>BMI: 23.8<br>Height: 166 cm<br>Mean weight: 65.6 kg<br><br>Height info: http://www.pisa2006.helsinki.fi/finland/statistics/statistics.htm<br><br>Weight information source ((31))

Study	Frequency	Duration	Intensity	Assigned MET h/week	MMET h/week	Additional information
**MONICA/KORA Augsburg Cohort Study** (9)		<1 h/week = Mean of 30 min/week<br><br>4 LTPA categories:<br>• "No activity" = No sports in summer or winter<br>• "Low activity" = Irregular, <1 h/week in <1 season<br>• "Moderate" = Regular 1 h/week in < 1 season<br>•"High" = regular >2 h/week in all seasons	MVPA = 4.5 MET	0<br>2.25<br>4.5<br>11.25	0<br>1.75<br>3.5<br>8.75	
**Health Professional's Follow-up Study** (10)				MET h/week LTPA :<br>• 0–5.9 (median=2.7)<br>• 6 –13.7 (median=9.6)<br>• 13.8–24.2 (median=18.6)<br>• 24.3–40.8 (median = 31.6)<br>• ≥40.9 (median=48.2)	2.1<br>7.47<br>14.47<br>24.56<br>37.45	
**The Pitt County Study** (14)	4 categories of LTPA<br>•"Inactive" = No strenuous PA, walking, home maintenance or gardening in a week.<br>•"Low" = > 15 min of home maintenance or gardening<br>•"Moderate" = Strenuous PA but not >3 times/week at 20 min/session<br>•"Strenuous" = Strenuous exercise/work >3/week and >20 min at a time	0 min<br>15 min of MVPA<br>60 min of VPA<br>105 min VPA	MVPA = 4.5 MET<br><br>VPA = 8 MET	0<br>1.125<br>8<br>14	0<br>0.875<br>7<br>12.25	

Study	Frequency	Duration	Intensity	Assigned MET h/week	MMET h/week	Additional information
**The Strong Heart Study** (15)			LTPA MET h/week:<br><br>• No activity<br>• <8 MET h<br>• 8 – 24 MET h<br>• >24 MET h	0<br>3.47<br>15.3<br>64.2	0<br>2.625<br>11.9<br>49.93	Mean MET h/ exposure category values were obtained upon correspondence with the authors
**Shanghai Women's Health Study** (12)		0<br>48 min<br>150 min<br>300 min	MET h/day/year	0<br>3.6<br>11.25<br>27	0<br>2.8<br>8.75<br>17.5	Additional data received from lead author
**English Longitudinal Study of Ageing** (16)	3 category index:<br><br>•Physical inactivity<br>•Low-intensity but not MVPA at least 1/week (at least 1/week = 2/week)<br>•MVPA at least 1/week (at least 1/week = 2/week)	45 min/session	Light intensity PA = 3 MET<br><br>MVPA = 4.5 MET	0<br>4.5<br>6.75	0<br>2.5<br>4.75	
**National Health Insurance Corporation Study** (17)		• "Inactive" (0 minutes/week)<br>• "Low" (1–149 min/week = 75 min)<br>• "Medium" (150–299 min/week) = 225 min)<br>• "High" (≥300 min/week) = 375 min	MVPA = 4.5 MET	0<br>5.625<br>16.875<br>28.125	0<br>4.375<br>13.125<br>21.875	

Study	Frequency	Duration	Intensity	Assigned MET h/week	MMET h/week	Additional information
**China Multicenter Collaborative Study of Cardiovascular Epidemiology (ChinaMUCA) and China CardiovascularHealth Study** (26)			Four PAL categories:<br><br>• Sedentary (PAL 1.00-1.39) =1.2<br>• Low active (PAL 1.40-1.59) = 1.5<br>•Active (PAL 1.60-1.89) = 1.75<br>•Very active (PAL >1.89) = 2.04	136.22<br>173.04<br>202.86<br>238.56	24.22<br>61.04<br>90.86<br>126.56	Males:<br>△PAL=∑(METn-1)×1.34×hn/24<br><br>Females:<br>△PAL=∑(METn-1)×1.42×hn/24<br>PAL=1.00＋△PAL<br><br>**PAL Level 1 = 1.2** 24*0.2/1.38784 = 3.46 MMET h/day *7=24.22 MMET h/week +(16 MET h*7) = 136.22 MET h/week<br><br>**PAL Level 2 = 1.5** 24*0.5/1.37664 = 8.72 MMET h/day *7=61.04 MMET h/week +(16 MET h*7) = 173.04 MET h/week<br><br>**PAL Level 3 = 1.75** 24*0.75/1.3864 = 12.98 MMET h/day *7=90.86 MMET h/week +(16 MET h*7) = 202.86 MET h/week<br><br>**PAL Level 4 = 2.04** 24*1.04/1.37992 = 18.08 MMET h/day *7=126.56 MMET h/week +(16 MET h*7) = 238.56 MET h/week<br><br>MET h dose calculations exclude 8h/day of sleep time
**Chin-Shan community cardiovascularcohort study (CCCC)]** (19)	Frequency of sports exercise:<br><br>• Never<br>• Rarely (30 min/week)<br>• Sometimes (2/week)<br>• Often (5/week)	45 min/session<br><br>• Never<br>• Rarely = (30 min/week)<br>• Sometimes = (2/week)<br>• Often = (5/week)	MVPA = 4.5 MET	0<br>2.25<br>6.75<br>16.875	0<br>1.75<br>4.75<br>13.125	
**Black Women's Health Study** (20)		• 0 h/week<br>• <1 h/week = 0.5 h<br>• 1–2 h/week = 1.5 h<br>• 3–4 h/week = 3.5 h<br>• 5–6 h/week = 5.5 h<br>• ≥7 h/week = 7.5 h	MVPA = 4.5 MET	0<br>2.25<br>6.75<br>15.75<br>20.25<br>33.75	0<br>1.75<br>5.25<br>12.25<br>15.75<br>26.25	

Study	Frequency	Duration	Intensity	Assigned MET h/week	MMET h/week	Additional information
**Kuopio Ischaemic Heart Disease Risk Factor Study** (13)	Frequency/month	1 h<br>8.8 h = 528 min<br>7.3 h = 438 min	4.1 MET/h<br>4.1 MET/h<br>6.4 MET/h	4.1<br>36.08<br>46.72	3.1<br>27.28<br>39.42	
**EPIC-InterAct** (25)		4 category index which incorporates OPA and LTPA:<br><br>• Inactive” = Sedentary job and no LTPA<br>• “Moderately inactive” = Sedentary job with 0.5 h LTPA/day or standing job with no LTPA<br>• “Moderately active” = Sedentary job with 0.5 -1 h LTPA/day or standing job with 0.5 h LTPA/day or physical job with no LTPA<br>• “Active” = Sedentary job with >1 h LTPA or standing job with .0.5 h LTPA or physical job with some LTPA or heavy manual job		0<br>9.966<br>20.04<br>33.4	0<br>8.216<br>14.79<br>24.65	Doses were assigned with the aid of findings from a validation study (32)

Study	Frequency	Duration	Intensity	Assigned MET h/week	MMET h/week	Additional information
**Honolulu Heart Program**(24)	Hours/week in each of 5 activity levels with 8 h of sleep per day excluded from the daily total	24 h PA index with 18 h of wake time	MVPA = 4.5 MET<br>Activity levels were multiplied by a weight based on mean oxygen consumption required to perform the activities of the category	129.85<br>153.3<br>168<br>187.25<br>300.3	17.85<br>41.3<br>56<br>75.25<br>188.2	Excl. 8 h per day as sleeping time<br><br>For MMET = Subtract 7x16 to account for 1 MET/h less per wake time
**Japanese male office worker cohort** (27)			Daily EE/kg for total PA:<br>•<33.1<br>(mid-point: 16.55)<br>•33.1–36.7<br>(mid-point: 34.9)<br>•36.8–40.3<br>(mid-point: 38.55)<br>•≥40.4 (mid-point: 42.15)	119.13<br>244.3<br>269.85<br>295.05	N/A	Mean Japanese weight:59.017 kg<br><br>Adjustment for male only cohort ~ +5 kg to the mean adult japanese weight. = Mean of 64 kg assumed for a Japanese office worker (33)<br><br>Level 1: 16.55*64=(1059.2 total PA EE/person/day)*7=7624.4 total PA EE/week<br><br>Level 2: 34.9*64=(2233.6 total PA EE/person/day)*7=15635.2 total PA EE/week<br><br>Level 3: 38.55*64=(2467.2 total PA EE/person/day)*7=17270.4 total PA EE/week<br><br>Level 4: 42.15*64=(2697.6 total PA EE/person/day)*7=18883.2 total PA EE/week<br><br>Conversion from EE/ week to MET h/ week:<br>PA EE/week/64=total PA MET h/week
**The Multiethnic Cohort (I)** (18)	PA frequency for strenuous sport was collapsed into 4 categories:<br>•Never<br>•0.5 – 1 h/week<br>•2 – 3 h/week<br>• >4h/week	•Never (= 0 min)<br>•0.5 – 1 h/week (45 min/week)<br>•2 – 3 h/week (150 min/week)<br>•>4h/week (=270 min/week)	MVPA = 4.5 MET	0<br>3.375<br>11.25<br>20.25	0<br>2.625<br>8.75<br>15.75	

Study	Frequency	Duration	Intensity	Assigned MET h/week	MMET h/week	Additional information
**The Multiethnic Cohort (II)** (18)	PA frequency for strenuous sport was collapsed into 4 categories:<br>•Never<br>•0.5 – 1 h/week<br>•2 – 3 h/week<br>• >4h/week	•Never (= 0 min)<br>•0.5 – 1 h/week (45 min/week)<br>•2 – 3 h/week (150 min/week)<br>•>4h/week (=270 min/week)	MVPA = 4.5 MET	0<br>3.375<br>11.25<br>20.25	0<br>2.625<br>8.75<br>15.75	
**Osaka Health Survey** (11)	3 categories of LTPA frequency on both weekdays and weekends:<br>•0<br>•1-2 (1.5 sessions)<br>• ≥3 (4.5 sessions)	45 min/session<br><br>0 min<br>67.5 min<br>202.5 min	MVPA = 4.5 MET	0<br>5.0625<br>15.1875	0<br>3.9375<br>11.8125	

MET h per week doses were assigned from descriptions identified within the individual studies or from correspondence with study authors.
(I)/(II) indicate sub-cohorts with independently reported risk estimates for diabetes type 2
CPA: Commuting physical activity; EE: Energy expenditure (in kcal); LTPA: Leisure time physical activity; MET: Metabolic Equivalent of Task; MMET: Marginal Metabolic Equivalent of Task, MET unit accounting for the individual basal metabolic rate; MVPA: Moderate to vigorous physical activity; OPA: Occupational physical activity; PA: Physical activity
Black font: Reported PA duration, frequency or intensity
Red: Assumed
Green: Calculated
Blue: Additional PA duration, frequency or intensity data received upon correspondence with the authors

ESM Table 3 Summary of MET h/week and MMET h/week sensitivity analysis dose assignment calculations for the prospective cohort studies included in the dose-response meta-analysis

Scenario A:

i. **Session duration:** 45 min
ii. **Intensity**: MVPA = 4.5 MET/h; LPA= 3 MET/h; VPA= 8 MET/h

Scenario B:

i. **Session duration:** 30 min
ii. **Intensity:** MVPA = 4.5 MET/h; LPA= 3 MET/h; VPA= 8 MET/h

Scenario C:

i. **Session duration**: 45 min
ii. **Intensity:** MVPA = 3.5 MET/h; LPA= 2 MET/h; VPA= 7 MET/h

Scenario D:

i. **Session duration**: 30 min
ii. **Intensity:** MVPA = 3.5 MET/h; LPA= 2 MET/h; VPA= 7 MET/h

Study	Scenario A: Assigned MET h/week	Scenario A: MMET h/week	Scenario B: Assigned MET h/week	Scenario B: MMET h/week	Scenario C: Assigned MET h/week	Scenario C: MMET h/week	Scenario D: Assigned MET h/week	Scenario D: MMET h/week
**Physicians Health Study** (1)	0 ID	0	0 D	0	0 I	0	0 ID	0
	1.6875	1.3125	1.125	0.875	1.3125	0.9375	0.875	0.625
	3.375	2.625	2.25	1.75	2.625	1.875	1.75	1.25
	10.125	7.875	6.75	5.25	7.875	5.625	3.5	3.75
	20.25	15.75	13.5	10.5	15.75	11.25	7	7.5
**Nurses' Health Study** (2) **(I)/(II)**	0 I	0	0	0	0 I	0	0 I	0
	1.125	0.875	1.125	0.875	0.875	0.625	0.875	0.625
	3.375	2.625	3.375	2.625	2.625	1.875	2.625	1.875
	7.875	6.125	7.875	6.125	6.125	4.375	6.125	4.375
	14.625	11.375	14.625	11.375	11.375	8.125	11.375	8.125

Study	Scenario A: Assigned MET h/week	Scenario A: MMET h/week	Scenario B: Assigned MET h/week	Scenario B: MMET h/week	Scenario C: Assigned MET h/week	Scenario C: MMET h/week	Scenario D: Assigned MET h/week	Scenario D: MMET h/week
**British Regional Heart Study** (3)	2.5 ID<br>3.375<br>6.75<br>11.25<br>16.875	1.75<br>2.625<br>5.25<br>8.75<br>13.125	1.125 D<br>2.25<br>5<br>7.25<br>11.25	0.875<br>1.75<br>3.5<br>5.25<br>8.75	1.75 I<br>2.625<br>5.25<br>8.75<br>13.125	1.25<br>1.875<br>3.75<br>6.25<br>9.375	0.875 ID<br>1.75<br>3.5<br>5.25<br>8.75	0.625<br>1.25<br>2.5<br>3.75<br>6.25
**Women's Health Study** (4)	1.37<br>4.1<br>10.34<br>22.69	1.04<br>3.2<br>8.04<br>17.65	1.37<br>4.1<br>10.34<br>22.69	1.04<br>3.2<br>8.04<br>17.65	1.37<br>4.1<br>10.34<br>22.69	1.04<br>3.2<br>8.04<br>17.65	1.37<br>4.1<br>10.34<br>22.69	1.04<br>3.2<br>8.04<br>17.65
**Eastern and South-Western Finnish adults** (5)	0<br>6.75<br>13.5	0<br>5.25<br>10.5	0<br>4.5<br>9	0<br>3.5<br>7	0<br>5.25<br>10.5	0<br>3.75<br>7.5	0<br>3.5<br>7	0<br>2.5<br>5
**Iowa Women's Health Study** (6)	0 ID<br>3.375<br>10.125<br>16.9	0<br>2.625<br>7.875<br>13.15	0 D<br>2.25<br>6.75<br>11.25	0<br>1.75<br>5.25<br>8.75	0 I<br>2.625<br>7.875<br>13.15	0<br>1.875<br>5.625<br>9.375	0 ID<br>1.75<br>5.25<br>8.75	0<br>1.25<br>3.75<br>6.25
**University of Pennsylvania Alumni** (7)	3.28<br>9.84<br>16.4<br>22.97<br>29.53<br>36.09<br>42.65<br>49.21	2.55<br>7.65<br>12.76<br>17.87<br>22.97<br>28.07<br>33.17<br>38.27	3.28<br>9.84<br>16.4<br>22.97<br>29.53<br>36.09<br>42.65<br>49.21	2.55<br>7.65<br>12.76<br>17.87<br>22.97<br>28.07<br>33.17<br>38.27	3.28<br>9.84<br>16.4<br>22.97<br>29.53<br>36.09<br>42.65<br>49.21	2.55<br>7.65<br>12.76<br>17.87<br>22.97<br>28.07<br>33.17<br>38.27	3.28<br>9.84<br>16.4<br>22.97<br>29.53<br>36.09<br>42.65<br>49.21	2.55<br>7.65<br>12.76<br>17.87<br>22.97<br>28.07<br>33.17<br>38.27
**North-Eastern Finnish Adult cohort** (8) **(I)**	6.8<br>18.5<br>28.3	5.3<br>14.4<br>22	6.8<br>18.5<br>28.3	5.3<br>14.4<br>22	6.8<br>18.5<br>28.3	5.3<br>14.4<br>22	6.8<br>18.5<br>28.3	5.3<br>14.4<br>22

Study	Scenario A: Assigned MET h/week	Scenario A: MMET h/week	Scenario B: Assigned MET h/week	Scenario B: MMET h/week	Scenario C: Assigned MET h/week	Scenario C: MMET h/week	Scenario D: Assigned MET h/week	Scenario D: MMET h/week
**North-Eastern Finnish Adult cohort** (8) **(II)**	6.9<br>18.3<br>25.7	5.4<br>14.2<br>20	6.9<br>18.3<br>25.7	5.4<br>14.2<br>20	6.9<br>18.3<br>25.7	5.4<br>14.2<br>20	6.9<br>18.3<br>25.7	5.4<br>14.2<br>20
**MONICA/KORA Augsburg Cohort Study** (9) **(I)**	0 ID<br>2.25<br>4.5<br>11.25	0<br>1.75<br>3.5<br>8.75	0 D<br>2.25<br>4.5<br>11.25	0<br>1.75<br>3.5<br>8.75	0 I<br>1.75<br>3.5<br>8.75	0<br>1.25<br>2.5<br>6.25	0 ID<br>1.75<br>3.5<br>8.75	0<br>1.25<br>2.5<br>6.25
**MONICA/KORA Augsburg Cohort Study** (9) **(II)**	0 ID<br>2.25<br>4.5<br>11.25	0<br>1.75<br>3.5<br>8.75	0 D<br>2.25<br>4.5<br>11.25	0<br>1.75<br>3.5<br>8.75	0 I<br>1.75<br>3.5<br>8.75	0<br>1.25<br>2.5<br>6.25	0 ID<br>1.75<br>3.5<br>8.75	0<br>1.25<br>2.5<br>6.25
**Health Professional's Follow-up Study** (34)	0<br>2.025<br>7.275<br>27	0<br>1.575<br>5.658<br>21	0<br>2.025<br>7.275<br>27	0<br>1.575<br>5.658<br>21	0<br>1.575<br>5.658<br>21	0<br>1.125<br>4.04<br>15	0<br>1.575<br>5.658<br>21	0<br>1.125<br>4.04<br>15
**The Pitt County Study** (14)	0 ID<br>1.125<br>8<br>14	0<br>0.875<br>7<br>12.25	0 D<br>1.125<br>8<br>14	0<br>0.875<br>7<br>12.25	0 I<br>0.875<br>7<br>12.25	0<br>0.625<br>6<br>10.5	0 ID<br>0.875<br>7<br>12.25	0<br>0.625<br>6<br>10.5
**The Strong Heart Study** (15)	0<br>3.47<br>15.3<br>64.2	0<br>2.625<br>11.9<br>49.93	0<br>3.47<br>15.3<br>64.2	0<br>2.625<br>11.9<br>49.93	0<br>3.47<br>15.3<br>64.2	0<br>2.625<br>11.9<br>49.93	0<br>3.47<br>15.3<br>64.2	0<br>2.625<br>11.9<br>49.93
**Shanghai Women's Health Study** (12)	0<br>3.6<br>11.25<br>27	0<br>2.8<br>8.75<br>17.5	0<br>3.6<br>11.25<br>27	0<br>2.8<br>8.75<br>17.5	0<br>3.6<br>11.25<br>27	0<br>2.8<br>8.75<br>17.5	0<br>3.6<br>11.25<br>27	0<br>2.8<br>8.75<br>17.5

Study	Scenario A: Assigned MET h/week	Scenario A: MMET h/week	Scenario B: Assigned MET h/week	Scenario B: MMET h/week	Scenario C: Assigned MET h/week	Scenario C: MMET h/week	Scenario D: Assigned MET h/week	Scenario D: MMET h/week
**English Longitudinal Study of Ageing** (16)	0 ID<br>3.375<br>7.03125	0<br>2.25<br>5.90625	0 D<br>3.375<br>4.6875	0<br>2.625<br>3.9375	0 I<br>2.25<br>5.90625	0<br>1<br>4.71	0 ID<br>1.5<br>3.9375	0<br>1<br>3.1875
**National Health Insurance Corporation Study** (17)	0 I<br>5.625<br>16.875<br>28.125	0<br>4.375<br>13.125<br>21.875	0<br>5.625<br>16.875<br>28.125	0<br>4.375<br>13.125<br>21.875	0 I<br>4.375<br>13.125<br>21.875	0<br>3.125<br>9.375<br>15.625	0 I<br>4.375<br>13.125<br>21.875	0<br>3.125<br>9.375<br>15.625
**Chin-Shan community cardiovascular cohort study (CCCC)**(19)	0 ID<br>2.25<br>6.75<br>16.875	0<br>1.75<br>4.75<br>13.125	0 D<br>2.25<br>4.5<br>11.25	0<br>1.75<br>3.5<br>8.75	0 I<br>1.75<br>4.75<br>13.125	0<br>1.25<br>3.75<br>9.375	0 I<br>1.75 D<br>3.5<br>8.75	0<br>1.25<br>2.5<br>6.25
**Black Women's Health Study** (20)	0 I<br>2.25<br>6.75<br>15.75<br>20.25<br>33.75	0<br>1.75<br>5.25<br>12.25<br>15.75<br>26.25	0<br>2.25<br>6.75<br>15.75<br>20.25<br>33.75	0<br>1.75<br>5.25<br>12.25<br>15.75<br>26.25	0<br>1.75<br>5.25<br>12.25<br>15.75<br>26.25	0<br>1.25<br>3.75<br>8.75<br>13.75<br>18.75	0<br>1.75<br>5.25<br>12.25<br>15.75<br>26.25	0<br>1.25<br>3.75<br>8.75<br>13.75<br>18.75
**Kuopio Ischaemic Heart Disease Risk Factor Study** (13)	4.1<br>36.08<br>46.72	3.1<br>27.28<br>39.42	4.1<br>36.08<br>46.72	3.1<br>27.28<br>39.42	4.1<br>36.08<br>46.72	3.1<br>27.28<br>39.42	4.1<br>36.08<br>46.72	3.1<br>27.28<br>39.42
**The Multiethnic Cohort (I)** (18)	0 I<br>3.375<br>11.25<br>20.25	0<br>2.625<br>8.75<br>15.75	0<br>3.375<br>11.25<br>20.25	0<br>2.625<br>8.75<br>15.75	0 I<br>2.625<br>8.75<br>15.75	0<br>1.875<br>6.25<br>11.25	0 I<br>2.625<br>8.75<br>15.75	0<br>1.875<br>6.25<br>11.25
**The Multiethnic Cohort (II)** (18)	0 I<br>3.375<br>11.25<br>20.25	0<br>2.625<br>8.75<br>15.75	0<br>3.375<br>11.25<br>20.25	0<br>2.625<br>8.75<br>15.75	0 I<br>2.625<br>8.75<br>15.75	0<br>1.875<br>6.25<br>11.25	0 I<br>2.625<br>8.75<br>15.75	0<br>1.875<br>6.25<br>11.25

Study	Scenario A: Assigned MET h/week	Scenario A: MMET h/week	Scenario B: Assigned MET h/week	Scenario B: MMET h/week	Scenario C: Assigned MET h/week	Scenario C: MMET h/week	Scenario D: Assigned MET h/week	Scenario D: MMET h/week
**Osaka Health Survey**(11)	0 ID<br>5.0625<br>15.1875	0<br>3.9375<br>11.8125	0 D<br>3.375<br>10.125	0<br>2.625<br>7.875	0 I<br>3.9375<br>11.8125	0<br>2.8125<br>8.4375	0 ID<br>2.625<br>7.875	0<br>1.875<br>5.625
**The Australian Diabetes, Obesity and Lifestyle Study** (21)	0 I<br>5.625<br>14.625	0<br>4.375<br>11.375	0<br>5.625<br>14.625	0<br>4.375<br>11.375	0 I<br>4.375<br>11.375	0<br>3.125<br>8.125	0 I<br>4.375<br>11.375	0<br>3.125<br>8.125
**The Nord-Trøndelag Health Survey** (22)	0 ID<br>1.6875<br>3.375<br>8.4375<br>23.625	0<br>1.3125<br>2.625<br>6.5625<br>18.375	0 D<br>1.125<br>2.25<br>5.625<br>15.75	0<br>0.875<br>1.75<br>4.375<br>12.25	0 I<br>1.3125<br>2.625<br>6.5625<br>18.375	0<br>0.9375<br>1.875<br>4.6875<br>13.125	0 ID<br>0.875<br>1.75<br>4.375<br>12.25	0<br>0.625<br>1.25<br>3.125<br>8.75
**45 and Up study** (23)	5.625 I<br>16.875<br>28.125	4.375<br>13.125<br>21.875	5.625 I<br>16.875<br>28.125	4.375<br>13.125<br>221.875	4.375 I<br>13.125<br>21.875	3.125<br>9.375<br>15.625	4.375 I<br>13.125<br>21.875	3.125<br>9.375<br>15.625
**Nr of studies to which assumptions had to be applied for:**	**15/27**		**9/27[a]**		**15/27[a]**		**15/27[a]**	
**Intensity**<br>**Duration**	**15/27**<br>**9/27**		**0/27**<br>**9/27**		**15/27**<br>**0/27**		**15/27**<br>**9/27**	

[a] Number of studies for which assumptions had to be adjusted relative to baseline assumptions made for LTPA dose harmonization in Scenario A MET h/week doses were assigned from descriptions identified within the individual studies or from correspondence with study authors.
(I)/(II) indicate sub-cohorts with independently reported risk estimates for diabetes type 2.
LPA: Low intensity physical activity; MET: Metabolic Equivalent of Task; MMET: Marginal Metabolic Equivalent of Task MET; PA: Physical activity; MVPA: Moderate to vigorous physical activity; VPA: Vigorous physical activity
Black font: Reported PA duration, frequency or intensity
Red: Assumed
Green: Calculated
Blue: Additional PA duration, frequency or intensity data received upon correspondence with the authors

ESM Table 4 Summary of the characteristics of 28 prospective cohort studies that investigate the association between levels of PA and incident type 2 diabetes, identified in the systematic literature search

Study	Country; study name	Cohort size	Sex	Age at baseline (years)	Follow-up (years)	% Cumulative incidence (cases/cohort)	Method of PA assessment	PA unit	PA assessment (PA dose in MET h/week[a])	Reported OR/RR/HR (95% CI)	Comments	Adjustments
Helmrich et al, 1991 (7)	USA; University of Pennsylvania Alumni	5990	M	39–68	14	3.4 (202/5990)	Paffenberger Physical Activity Index/College Alumnus Questionnaire (PAI-CAQ)[b]	Weekly EE for LTPA	Weekly total EE for LTPA in 500 kcal (2092 kJ) increments:<br>• <500 (3.3)<br>• 500–999 (9.5)<br>• 1000–1499 (16.4)<br>• 1500–1999 (23.0)<br>• 2000–2499 (29.5)<br>• 2500–2999 (36.1)<br>• 3000–3499 (42.7)<br>• ≥3000 (49.2)	RR for T2D relative to most inactive group:<br>1.00<br>0.94 (0.9, 0.98)<br>0.79<br>0.78<br>0.68<br>0.90<br>0.86<br>0.52		Age
Burchfiel et al, 1995 (24)	USA; Honolulu Heart Program[c]	6815	M	45–68	6	5.7 (391/6815)	Questionnaire	H/week in each of five activity levels (multiplied by a weight based on mean oxygen consumption required to perform the activities of the category)	Composite score based on 24 h PA dose calculated by summing the hours spent in each activity intensity level and multiplying by a respective weight factor and categorised into quintiles:<br>• 24.1–29.0 (129.9)<br>• 29.1–30.7 (153.3)<br>• 30.8–33.2 (168.0)<br>• 33.3–36.2 (187.3)<br>• 36.3–65.5 (300.3)	OR for T2D relative to most inactive group:<br>1.00<br>0.86 (0.64, 1.16)<br>0.81 (0.60, 1.09)<br>0.72 (0.53, 1.03)<br>0.47 (0.33, 0.67)	Cohort of middle-aged Japanese-American men	Age
Lynch et al, 1996 (13)	Finland; Kuopio Ischaemic Heart Disease Risk Factor Study[c]	2682	M	42–60	18	23.9 (640/2682)	Minnesota LTPA Questionnaire [b] (modified)	Frequency/month; intensity was estimated on a scale of 1 (lowest) –3 (highest)	1 year retrospective leisure time physical activity assessment of 15 common PA types:<br>• low PA (4.1)<br>• PA below an intensity of 5.5 MET but >2 h/week (36.1)<br>• PA (>5.5 MET intensity) >40 min/week (46.7)	PA (>5.5 MET intensity) >40 min/week had an OR of 0.83 (0.63, 1.10) for T2D compared with participants reporting less duration/intensity of PA.<br><br>The OR for T2D observed for PA below an intensity of 5.5 MET but >2 h/week was 0.83 (0.66, 1.03)		Age, fasting baseline glucose, serum triacylglycerol, BP, parental history of diabetes, alcohol consumption, BMI
Haapanen et al, 1997 (8)	Finland; North-Eastern Finnish Adult cohort (I)	1340	M	35–63[d]	10	4.8 (64/1340)	Self-administered questionnaire	LTPA EE/week Frequency of vigorous PA/week	Weekly LTPA EE (kcal) categories for men:<br><br>• low: 0–1100 (6.8)<br>• moderate: 1101–1900 (18.5)<br>• high: >1900 (28.3)<br><br>Participants were asked to report average frequency of vigorous activity (≥6 MET) as:<br>• ≥1/week<br>• <1/week	RR for T2D relative to most inactive group:<br>1.00<br>1.54 (0.83, 2.84)<br>1.63 (0.92, 2.88)	Risk estimates were reversed to set the lowest level of LTPA as the referent category using the Hamling method (35)	Age

Study	Country; study name	Cohort size	Sex	Age at baseline (years)	Follow-up (years)	% Cumulative incidence (cases/cohort)	Method of PA assessment	PA unit	PA assessment (PA dose in MET h/week[a])	Reported OR/RR/HR (95% CI)	Comments	Adjustments
Haapanen et al, 1997 (8)	Finland; North-Eastern Finnish Adult cohort (II)	1500	F	35–63[d]	10	3.6 (54/1500)	Self-administered questionnaire	LTPA EE/week<br>Frequency of vigorous PA/week	Weekly LTPA EE (kcal) categories for women:<br>• low: 0–900 (6.9)<br>• moderate: 901–1500 (18.3)<br>• high: >1500 (25.7)<br><br>Participants were asked to report average frequency of vigorous activity (≥6 MET) as:<br>• ≥1/week<br>• <1/week	RR for T2D relative to most inactive group:<br>1.00<br>2.64 (1.28, 5.44)<br>2.23 (0.95, 5.23)	Risk estimates were reversed to set the lowest level of LTPA as the referent category using the Hamling method (35)	Age
James et al, 1998 (14)	USA; Pitt County Study	916	M/F	25–55	5	8.5 (78/916)	Questionnaire	Physical activity index based on frequency of physical activity enough to work up a sweat and result in heavy breathing<br>Frequency of strenuous work/exercise >20 min at a time	Four categories of LTPA level defined as:<br>• 'inactive' = individuals who did not report any strenuous work/exercise, walking, home maintenance or gardening in a week. (0)<br>• 'low' = some home maintenance work (>15 min) or gardening during an average week (1.1)<br>• 'moderate' = some strenuous work/exercise but not >3 times/week at 20 min/session (4.5)<br>• 'strenuous' = strenuous exercise/work >3/week and >20 min at a time (7.9)	OR for T2D relative to most inactive group:<br>1.00<br>0.51 (0.20, 1.28)<br>0.35 (0.12, 0.98)<br>0.65 (0.26, 1.63)	Cohort exclusively consisted of individuals of African-American ethnicity	Age, sex, education, WHR, BMI
Folsom et al, 2000 (6)	USA; Iowa Women's Health Study	34,257	F	55–69	12	5.8 (1997/34,257)	Mailed questionnaire<br>Standard questions from the Gallup poll with the addition of questions specific to LTPA	Frequency/week	Initial assessment of any habitual PA (Y/N)<br>Participants reporting regular PA needed to specify frequency of moderate and vigorous PA (>6 MET) which was categorised into quartiles:<br>• rare or never (0)<br>• 1/week–few times/month (3.4)<br>• 2–4/week (10.1)<br>• >4/week (16.9)	RR for T2D relative to most inactive group:<br>1.00<br>0.80 (0.71, 0.90)<br>0.65 (0.58, 0.74)<br>0.51 (0.43, 0.59)<br><br>BMI-adjusted RR for T2D relative to most inactive group:<br>1.00<br>0.90 (0.79, 1.01)<br>0.86 (0.76, 0.98)<br>0.73 (0.62, 0.85)	Cohort consisted exclusively of postmenopausal women	Age, education, smoking, alcohol intake, oestrogen replacement, energy intake, wholegrain intake, dietary score, family history of diabetes (+ BMI and WHR in further adjusted model)

Study	Country; study name	Cohort size	Sex	Age at baseline (years)	Follow-up (years)	% Cumulative incidence (cases/cohort)	Method of PA assessment	PA unit	PA assessment (PA dose in MET h/week[a])	Reported OR/RR/HR (95% CI)	Comments	Adjustments
Okada et al, 2000 (11)	Japan; Osaka Health Survey	6013	M	35–60	10	7.4 (444/6013)	Questionnaire	Min/week during the week or weekend	Three categories of weekly LTPA:<br>• 'sedentary': no regular exercise (0)<br>• 'moderate': 1 h/week (5.1)<br>• 'vigorous': ≥1 h/week exercise 'enough to work up a sweat' (15.2)	RR for T2D relative to most inactive group:<br>1.00<br>0.65 (0.45, 0.95)<br>0.52 (0.35, 0.79)<br><br>BMI-adjusted RR for T2D relative to most inactive group:<br>1.00<br>0.80 (0.71, 0.99)<br>0.55 (0.34, 0.87)		Age, daily alcohol consumption, smoking habits, BP levels, parental history of T2D (+ BMI in the BMI-adjusted model)
Wannamethee et al, 2000 (3)	UK; British Regional Heart Study	7735	M	40–59	16.8	2.5 (196/7735)	Minnesota LTPA Questionnaire in a nurse-administered face to face interview[b]	Weekly frequency of three intensity categories (combined to an overall PA score):<br>(1) regular walking and cycling<br>(2) recreational activity e.g. gardening or pleasure walking<br>(3) sporting activity e.g. running, golf, swimming or tennis	A PA score was calculated depending on dose/type of regular exercise. Scores were categorised into five groups:<br>• 'inactive/occasional' (irregular walking or recreational activity) (2.5)<br>• 'light' (more frequent recreational activities or exercise <1/week, or regular walking + recreational activity) (3.4)<br>• 'moderate' (frequent weekend recreational activities + regular walking, or sporting activity 1/week) (6.8)<br>• 'moderately vigorous' (exercise 1/week or frequent cycling + recreational activities or walking or frequent sporting activities) (11.3)<br>• 'vigorous' (very frequent exercise, or frequent exercise + recreational activities) (16.9)	RR for T2D relative to most inactive group:<br>1.00<br>0.65 (0.42, 1.00)<br>0.60 (0.38, 0.95)<br>0.42 (0.24, 0.72)<br>0.36 (0.21, 0.62)<br><br>BMI-adjusted RR for T2D relative to most inactive group:<br>1.00<br>0.66 (0.42, 1.02)<br>0.65 (0.41, 1.03)<br>0.48 (0.28, 0.83)<br>0.46 (0.27, 0.79)		Age, smoking, alcohol, social class, pre-existing CHD (+ BMI in the BMI-adjusted model)
Hu et al, 2004 (5)	Finland; Eastern and South-Western Finnish adults	4369	M/F	45–64	9.4	2.8 (120/4369)	Mailed questionnaire	Min/week	A simplified index for LTPA scores was derived and reported in three categories:<br>• 'low' = light levels of occupational, commuting (<30 min) and inactive LTPA (0)<br>• 'moderate' = 1 type of LTPA activity/week (3.4)<br>• 'high' = 2 or 3 types of LTPA/week (8.4)	RR for T2D relative to most inactive group:<br>1.00<br>0.71 (0.46, 1.12)<br>0.32 (0.19, 0.56)<br>BMI-adjusted RR for T2D relative to most inactive group:<br>1.00<br>0.85 (0.54, 1.34)<br>0.43 (0.25, 0.74)		Age, study year, sex, systolic BP, smoking, education (+ BMI in the BMI-adjusted model)

Study	Country; study name	Cohort size	Sex	Age at baseline (years)	Follow-up (years)	% Cumulative incidence (cases/cohort)	Method of PA assessment	PA unit	PA assessment (PA dose in MET h/week[a])	Reported OR/RR/HR (95% CI)	Comments	Adjustments
Nakanishi et al, 2004 (27)	Japan; Japanese male office worker cohort	2924	M	35–59	7	5.8 (168/2924)	Self-administered 1 day activity record	Daily EE for total PA	Quartiles of daily EE/kg for 20 activities:<br>• <33.1 (119.1)<br>• 33.1–36.7 (244.3)<br>• 36.8–40.3 (269.9)<br>• ≥40.4 (295.0)	RR for T2D relative to most inactive group:<br>1.00<br>0.65 (0.45, 0.95)<br>0.52 (0.35, 0.79)<br>0.27 (0.16, 0.45)<br><br>BMI-adjusted RR for T2D relative to most inactive group:<br>1.00<br>0.76 (0.52, 1.11)<br>0.70 (0.46, 1.06)<br>0.41 (0.24, 0.70)		Age, family history of diabetes, alcohol consumption, cigarette smoking, weekly EE on PA, systolic BP, HDL-cholesterol and triacylglycerol at baseline (+ BMI in the BMI-adjusted model)
Weinstein et al, 2004 (4)	USA; Women's Health Study	37,878	F	55	6.9	3.6 (1361/37,878)	The NHS II Physical Activity & Inactivity Questionnaire	LTPA EE/week and min/week walking	EE/week (kcal) for LTPA in categories of:<br>• 0–199 (1.4)<br>• 200–599 (4.1)<br>• 600–1499 (10.3)<br>• >1500 (22.7)	HR for T2D relative to most inactive group:<br>1.00<br>0.78 (0.68, 0.90)<br>0.69 (0.59, 0.80)<br>0.74 (0.63, 0.88)<br><br>BMI-adjusted HR for T2D relative to most inactive group:<br>1.00<br>0.91 (0.79, 1.06)<br>0.86 (0.74, 1.01)<br>0.82 (0.70, 0.97)		Age, family history of diabetes, smoking, alcohol, hormone therapy, hypertension, high cholesterol, dietary factors, randomised treatment group within the Women's Health Study (+ BMI in the BMI-adjusted model)
Hsia et al, 2005 (36)	USA; Women's Health Initiative[e]	87,907	F	63.8	5.1	2.6 (2271/87,907)	Women's Health Initiative Questionnaire[b]	MET h/week	Categories of weekly MET h for total physical activity:<br>• 0–2.2 (1.2)<br>• 2.3–7.4 (4.9)<br>• 7.5–13.9 (10.7)<br>• 14.0–23.4 (18.7)<br>• >23.4 (28.1)	RR for T2D relative to most inactive group:<br>1.00<br>0.91 (0.80, 1.03)<br>0.80 (0.70, 0.91)<br>0.86 (0.75, 0.99)<br>0.78 (0.67, 0.91)	Ethnically diverse population of white, African-American, Hispanic, American Indian and Asian postmenopausal women	Age, BMI, alcohol, education, smoking, hypertension, hypercholesterolaemia, dietary fibre intake, percent energy from carbohydrate
Meisinger et al, 2005 (9)	Germany; MONICA/KORA Augsburg Cohort Study (I)	4069	M	24–75[d]	7.4	3.6 (145/4069)	Face-to-face interview	H/week Frequency/season (summer/winter)	Four categories of LTPA defined as:<br>• 'no activity' = no sports in summer or winter (0)<br>• 'low activity' = irregular, <1 h/week in at least one season (2.3)<br>• 'moderate' = regular 1 h/week in at least one season (4.5)<br>• 'high' = regular > 2 h/week in both seasons (11.3)	HR for T2D relative to most inactive group:<br>1.00<br>0.86 (0.57, 1.29)<br>0.73 (0.45, 1.20)<br>0.73 (0.45, 1.20)		Age, survey, actual hypertension, dyslipidaemia, parental history of diabetes, regular smoking, alcohol intake, education, BMI

Study	Country; study name	Cohort size	Sex	Age at baseline (years)	Follow-up (years)	% Cumulative incidence (cases/cohort)	Method of PA assessment	PA unit	PA assessment (PA dose in MET h/week[a])	Reported OR/RR/HR (95% CI)	Comments	Adjustments
Meisinger et al, 2005 (9)	Germany; MONICA/KORA Augsburg Cohort Study (II)	4034	F	24–75[d]	7.4	2 (82/4034)	Face-to-face interview	H/week Frequency/season (summer/winter)	Four categories of LTPA defined as:<br>• 'no activity' = no sports in summer or winter (0)<br>• 'low activity' = irregular, <1 h/week in at least one season (2.3)<br>• 'moderate' = regular 1 h/week in at least one season (4.5)<br>• 'high' = regular, >2 h/week in both seasons (11.3)	HR for T2D relative to most inactive group:<br>1.00<br>0.85 (0.51, 1.41)<br>0.59 (0.31, 1.11)<br>0.21 (0.05, 0.86)		Age, survey, actual hypertension, dyslipidaemia, parental history of diabetes, regular smoking, alcohol intake, education, BMI
Villegas et al, 2006 (12)	China; Shanghai Women's Health Study	70,658	F	40–70	4.6	2.8 (1973/70,658)	Face-to-face interview at baseline and the Shanghai Women's Health Study Physical Activity Questionnaire (SWHS-PAQ)[b]	MET h/day/year	EE for retrospective regular LTPA during previous 5 years in MET h/day/year, DPA (including walking), CPA (bus or vehicle, walking or cycling <30 min/day or 30+ min/day) and EE in OPA (high/medium/low)<br><br>LTPA h/day:<br>• 0 (0)<br>• <0.8 (3.6)<br>• 0.8–1.99 (11.3)<br>• >1.99 (27.0)	RR for T2D relative to most inactive group:<br>1.00<br>0.89 (0.76, 1.03)<br>0.99 (0.85, 1.15)<br>0.83 (0.70, 0.97)		Age, daily calories, education level, income level, occupation, smoking, alcohol, hypertension, chronic diseases
Carlsson et al, 2007 (22)	Sweden; Nord-Trøndelag Health Survey	38,800	M/F	≥20	11	1.9 (738/38,800)	Questionnaire	Exercise frequency ranging from 'never' to 'every day'	Frequency of weekly LTPA:<br>• never (0)<br>• <1×/week (1.7)<br>• 1×/week (3.4)<br>• 2–3×/week (8.4)<br>• every day (23.6)	RR for T2D relative to most inactive group:<br>1.00<br>0.79 (0.64, 0.99)<br>0.61 (0.48, 0.77)<br>0.60 (0.48, 0.73)<br>0.49 (0.37, 0.66)	Risk estimates were reversed to set the lowest level of LTPA as the referent category using the Hamling method (35)	Sex, smoking, BMI
Magliano et al, 2008 (21)	Australia; The Australian Diabetes, Obesity and Lifestyle Study	5842	M/F	50.9	5	3.8 (224/5842)	Interviewer-administered Active Australia questionnaire	Total LTPA time derived from sum of the time spent performing MVPA + double the time spent performing VPA in the previous week	Categories of weekly LTPA min/week:<br>• inactive (0 min/week)<br>• insufficient (1–49 min/week) (5.6)<br>• sufficient (≥150 min/week) (14.6)	OR for T2D relative to most inactive group:<br>1.00<br>0.97 (0.58, 1.63)<br>0.64 (0.46, 0.89)<br><br>BMI-adjusted OR for T2D relative to most inactive group:<br>1.00<br>0.86 (0.58, 1.27)<br>0.50 (0.35, 0.72)	Risk estimates were reversed to set the lowest level of LTPA as the referent category using the Hamling method (35)	Age, sex, waist circumference, smoking, education, hypertension, family history of diabetes, log FPG, hypertriacylglycerolaemia, low HDL-cholesterol and cholesterol

Study	Country; study name	Cohort size	Sex	Age at baseline (years)	Follow-up (years)	% Cumulative incidence (cases/cohort)	Method of PA assessment	PA unit	PA assessment (PA dose in MET h/week[a])	Reported OR/RR/HR (95% CI)	Comments	Adjustments
Chien et al, 2009 (19)	Taiwan; Chin-Shan community cardiovascular cohort study (CCCC)	1639	M/F	>35	9.02	19 (312/1639)	Baecke Questionnaire of Habitual Physical Activity[b]	Sports, occupational and leisure PA frequency was rated on a 5-point Likert scale	Frequency of sports exercise was reported in quartiles corresponding to:<br>• never (0)<br>• rarely (2.3)<br>• sometimes (6.8)<br>• often (16.9)	RR for T2D relative to most inactive group:<br>1.00<br>0.83 (0.62, 1.12)<br>0.70 (0.52, 0.94)<br>0.74 (0.54, 1.03)<br><br>BMI-adjusted RR for T2D relative to most inactive group:<br>1.00<br>0.82 (0.60, 1.12)<br>0.65 (0.47, 0.89)<br>0.68 (0.49, 0.95)		Age, sex, the metabolic syndrome, smoking, current alcohol drinking, marital status, education level, occupation, hypertension status, HDL-cholesterol, triacylglycerols, glucose levels, family history of diabetes, BMI
Fretts et al, 2009 (15)	USA; The Strong Heart Study	1651	M/F	45–74	10	27.5 (454/1651)	Questionnaire	LTPA<br>MET h/week<br>Total PA<br>MET h/week	LTPA MET h/week:<br>• no activity<br>• <8 MET h/week (3.5)<br>• 8–24 MET h/week (15.3)<br>• >24 MET h/week (64.2)<br><br>Total PA MET h/week:<br><br>• no activity<br>• <30 MET h/week<br>• 30–106 MET h/week<br>• >106 MET h/week	OR for T2D relative to most inactive group:<br>1.00<br>1.04 (0.74, 1.47)<br>0.76 (0.55, 1.07)<br>0.68 (0.49, 0.95)<br><br>BMI-adjusted OR for T2D relative to most inactive group:<br>1.00<br>1.09 (0.76, 1.56)<br>0.80 (0.56, 1.15)<br>0.75 (0.53, 1.00)	Cohort consisted exclusively of individuals of American Indian ethnicity	Age, study site, sex, education, cigarette smoking, alcohol use, family history of diabetes, systolic BP, diastolic BP, HDL-cholesterol, LDL-cholesterol, plasma fibrinogen, BMI
Krishnan et al, 2009 (20)	USA; Black Women's Health Study	45,668	F	21–69	10	6.4 (2928/45,668)	Questionnaire[b]	H/week spent on VPA (e.g. running, swimming), walking for exercise and walking to and from work	MVPA was reported in categories of:<br>• 0 h/week (0)<br>• <1 h/week (2.3)<br>• 1–2 h/week (6.8)<br>• 3–4 h/week (15.8)<br>• 5–6 h/week (20.3)<br>• ≥7 h/week (33.8)	RR for T2D relative to most inactive group:<br>1.00<br>0.90 (0.82, 0.99)<br>0.77 (0.69, 0.85)<br>0.53 (0.45, 0.63)<br>0.49 (0.38, 0.64)<br>0.43 (0.31, 0.59)		Age, time period, family history of diabetes, years of education, family income, marital status, cigarette use, alcohol use, energy intake, coffee consumption, television watching, walking

Study	Country; study name	Cohort size	Sex	Age at baseline (years)	Follow-up (years)	% Cumulative incidence (cases/cohort)	Method of PA assessment	PA unit	PA assessment (PA dose in MET h/week[a])	Reported OR/RR/HR (95% CI)	Comments	Adjustments
Siegel et al, 2009 (1)	USA; Physicians Health Study	20,757	M	40–84	23.1	8.8 (1836/20,757)	Paffenberger Physical Activity Index/College Alumnus Questionnaire (PAI-CAQ)[b]	Weekly frequency of vigorous exercise 'enough to work up a sweat'	Weekly vigorous exercise in number of times/week:<br>• rarely/never (0)<br>• 1–3/month (1.7)<br>• once/week (3.4)<br>• 2–4/week (10.1)<br>• ≥5 times/week (20.3)	RR for T2D relative to most inactive group:<br>1.00<br>0.84 (0.72, 0.98)<br>0.78 (0.68, 0.91)<br>0.63 (0.55, 0.73)<br>0.49 (0.41, 0.59)<br><br>BMI-adjusted RR for T2D relative to most inactive group:<br>1.00<br>0.84 (0.72, 0.98)<br>0.81 (0.70, 0.93)<br>0.69 (0.61, 0.79)<br>0.58 (0.48, 0.69)		Age, alcohol use, smoking, history of high cholesterol, history of hypertension (+ BMI in the BMI-adjusted model)
Demakakos et al, 2010 (16)	UK; English Longitudinal Study of Ageing (ELSA)	7466	M/F	62.9–68.3	3.8	3.5 (258/7466)	Questionnaire	Frequency/week	Frequency of each vigorous, moderate and low intensity PA:<br>• >1/week<br>• 1/week<br>• 1–3/month<br>• Hardly ever/never<br><br>Combined to a derived summary three-category index:<br>• physical inactivity (0)<br>• low-intensity but not vigorous/moderate-intensity physical activity at least once a week (3.4)<br>• MVPA or VPA at least once a week (7.0)	HR for T2D relative to most inactive group:<br>1.00<br>0.76 (0.51, 1.13)<br>0.49 (0.33, 0.71)<br><br>BMI-adjusted HR for T2D relative to most inactive group:<br>1.00<br>0.83 (0.56, 1.23)<br>0.57 (0.39, 0.84)		Age, age-squared, sex, marital status, educational attainment, total household wealth (+ BMI in the BMI-adjusted model)
Ekelund et al, 2012 (25)	Denmark, France, Germany, Italy, Spain, Sweden, UK and the Netherlands ; EPIC-InterAct (I)	EPIC total cohort 340,234; InterAct subcohort 15,934; men 6009	M/F; M/F; M	51.4–55.4 (mean)	12.3	3.6 (12,403/340,234); 4.9 (778/15,934); 6.5 (391/6009)	EPIC Physical Activity Questionnaire including questions specific to LTPA	Physical activity index (including OPA)	Four category index which incorporates OPA and LTPA:<br>• 'inactive' = sedentary job and no LTPA (0)<br>• 'moderately inactive' = sedentary job with 0.5 h LTPA/day or standing job with no LTPA (10.0)<br>• 'moderately active' = sedentary job with 0.5–1 h LTPA/day or standing job with 0.5 h LTPA/day or	HR for T2D relative to most inactive group:<br>1.00<br>0.89 (0.78, 1.01)<br>0.73 (0.64, 0.85)<br>0.69 (0.60, 0.80)		Education, smoking status, alcohol consumption, energy intake, BMI

Study	Country; study name	Cohort size	Sex	Age at baseline (years)	Follow-up (years)	% Cumulative incidence (cases/cohort)	Method of PA assessment	PA unit	PA assessment (PA dose in MET h/week[a])	Reported OR/RR/HR (95% CI)	Comments	Adjustments
									physical job with no LTPA (20.0)<br>• 'active' = sedentary job with >1 h LTPA or standing job with 0.5 h LTPA or physical job with some LTPA or heavy manual job (33.4)			
Ekelund et al, 2012 (25)	Denmark, France, Germany, Italy, Spain, Sweden, UK and the Netherlands; EPIC-InterAct (II)	EPIC total cohort 340,234; InterAct subcohort 15,934; women 9925	M/F; M/F; F	51.4–55.4 (mean)	12.3	3.6 (12,403/340,234); 4.9(778/15,934); 4(397/9925)	EPIC Physical Activity Questionnaire including questions specific to LTPA	Physical activity index (including OPA)	Four category index which incorporates OPA and LTPA:<br>• 'inactive' = sedentary job and no LTPA (0)<br>• 'moderately inactive' = sedentary job with 0.5 h LTPA/day or standing job with no LTPA (10.0)<br>• 'moderately active' = sedentary job with 0.5–1 h LTPA/day or standing job with 0.5 h LTPA/day or physical job with no LTPA (20.0)<br>• 'active' = sedentary job with >1 h LTPA or standing job with 0.5 h LTPA or physical job with some LTPA or heavy manual job (33.4)	HR for T2D relative to most inactive group:<br>1.00<br>0.93 (0.89, 0.98)<br>0.89 (0.78, 1.01)<br>0.79 (0.68, 0.91)		Education, smoking status, alcohol consumption, energy intake, BMI
Grøntved et al, 2012 (34)	USA; Health Professionals Follow-up Study	32,002	M	44–79	18	7.1 (2278/32,002)	Health Professionals Follow-Up Study Physical Activity Questionnaire & a biannual follow-up questionnaire[b]	Aerobic exercise min/week	Total time spent on aerobic exercise of at least moderate intensity (≥3 METs); participants grouped into four categories:<br>• none (0)<br>• 1–59 min (2.0)<br>• 60–149 min (7.3)<br>• ≥150 min (27.0)	RR for T2D relative to most inactive group:<br>1.00<br>0.93 (0.81, 1.06)<br>0.61 (0.60, 0.80)<br>0.55 (0.42, 0.55)<br><br>BMI-adjusted RR for T2D relative to most inactive group:<br>1.00<br>1.00 (0.88, 1.15)<br>0.80 (0.69, 0.92)<br>0.61 (0.53, 0.70)		Age, smoking, alcohol consumption, coffee intake, race, family history of diabetes, total energy, trans fat, polyunsaturated fat to saturated fat ratio, cereal fibre, wholegrain, and glycaemic load, weight, physical activity of at least moderate intensity, TV viewing (+ BMI in the BMI-adjusted model)

Study	Country; study name	Cohort size	Sex	Age at baseline (years)	Follow-up (years)	% Cumulative incidence (cases/cohort)	Method of PA assessment	PA unit	PA assessment (PA dose in MET h/week[a])	Reported OR/RR/HR (95% CI)	Comments	Adjustments
Lee et al, 2012 (17)	South Korea; National Health Insurance Corporation Study	675,496	M	39.4	7.5	7.9 (52,995/675,496)	Questionnaire	Frequency and duration of LTPA that 'causes sweating'	Physical activity volume was calculated and participants were classified into four categories:<br>• 'inactive' (0 min/week) (0)<br>• 'low' (1–149 min/week) (5.6)<br>• 'medium' (150–299 min/week) (16.9)<br>• 'high' (≥300 min/week) (28.1)	HR for T2D relative to most inactive group:<br>1.00<br>0.98 (0.96, 0.99)<br>0.94 (0.91, 0.96)<br>0.94 (0.91, 0.97)<br><br>BMI-adjusted HR for T2D relative to most inactive group:<br>1.00<br>0.95 (0.93, 0.97)<br>0.90 (0.87, 0.93)<br>0.91 (0.89, 0.94)		Age, smoking status, alcohol intake, hypertension, parental diabetes, baseline glucose (+ BMI in the BMI-adjusted model)
Steinbrecher et al, 2012 (18)	USA; The Multiethnic Cohort (I)	35,976 (men)	M	45–75	14	12.6 (4527/35,927)	Questionnaire	H/week of strenuous sport, vigorous work or moderate activity	Physical activity frequency for strenuous sport was collapsed into four categories:<br>• never (0)<br>• 0.5–1 h/week (3.4)<br>• 2–3 h/week (11.3)<br>• >4 h/week (20.3)	HR for T2D relative to most inactive group:<br>1.00<br>0.94 (0.87, 1.02)<br>0.85 (0.77, 0.94)<br>0.80 (0.72, 0.88)		Age, ethnicity, education, BMI
Steinbrecher et al, 2012 (18)	USA; The Multiethnic Cohort (II)	38,937 (women)	F	45–75	14	10.4 (4034/38,937)	Questionnaire	H/week of strenuous sport, vigorous work or moderate activity	Physical activity frequency for strenuous sport was collapsed into four categories:<br>• never (0)<br>• 0.5–1 h/week (3.4)<br>• 2–3 h/week (11.3)<br>• >4 h/week (20.3)	HR for T2D relative to most inactive group:<br>1.00<br>1.00 (0.91, 1.09)<br>0.85 (0.75, 0.96)<br>0.67 (0.57, 0.79)		Age, ethnicity, education, BMI
Shi et al, 2013 (37)	China; Shanghai Men's Health Study[e]	51,464	M	54.1	5.4	2.5 (1304/51,464)	The Shanghai Men's Health Study Physical Activity Questionnaire	Appraisal of LTPA, DPA and CPA Participants had to indicate whether they had undertaken any LTPA ≥1/week over the preceding 5 years	LTPA volume was reported as four categories of MET h/week/year:<br><br>• none (0)<br>• low (<1.2) (4.2)<br>• medium (1.2–3) (14.7)<br>• high (≥3) (27.3)	HR for T2D relative to most inactive group:<br>1.00<br>0.79 (0.65, 0.96)<br>0.87 (0.72, 1.04)<br>0.87 (0.75, 1.07)<br><br>Fully-adjusted HR for T2D relative to most inactive group:<br>1.00<br>0.80 (0.65, 0.97)<br>0.89 (0.74, 1.07)<br>0.91 (0.76, 1.08)		Age, energy intake, smoking, alcohol consumption, education level, occupation, income level, hypertension, family history of diabetes (+ BMI and WHR in fully-adjusted model)

Study	Country; study name	Cohort size	Sex	Age at baseline (years)	Follow-up (years)	% Cumulative incidence (cases/cohort)	Method of PA assessment	PA unit	PA assessment (PA dose in MET h/week[a])	Reported OR/RR/HR (95% CI)	Comments	Adjustments
Fan et al, 2015 (26)	China; China Multicenter Collaborative Study of Cardiovascular Epidemiology (China MUCA) and China Cardiovascular Health Study	6348	M/F	49.2	7.9	7.5 (478/6348)	Questionnaire	Physical activity level (PAL) = method to estimate total daily energy expenditure (38)	Average h/day spent in vigorous activity (e.g. jogging), moderate activity (e.g. yard work), light activity (e.g. office work), sedentary activity (e.g. TV) and periods of reclining during the previous 12 months<br><br>Four PAL categories:<br>• sedentary (PAL 1.00–1.39) (136.2)<br>• low active (PAL 1.40–1.59) (173.0)<br>• active (PAL 1.60–1.89) (202.9)<br>• very active (PAL >1.89) (238.6)	HR for T2D relative to most inactive group:<br>1.00<br>0.92 (0.69, 1.22)<br>0.70 (0.52, 0.93)<br>0.55 (0.42, 0.73)<br><br>BMI-adjusted HR for T2D relative to most inactive group:<br>1.00<br>0.82 (0.62, 1.09)<br>0.63 (0.47, 0.83)<br>0.47 (0.36, 0.61)	Urban residents only included in the analysis as the PAL questionnaire was not considered valid for rural participants	Age, sex, geographic region, educational level, cigarette smoking, alcohol consumption, family history of diabetes (+ BMI in the BMI adjusted model)
Grøntved et al, 2014 (2)	USA; Nurses' Health Study (2000–2008) (I)	51,642	F	53–81	8	4.2 (2158/51,642)	The NHS Physical Activity & Inactivity questionnaire with biannual postal questionnaires[b]	MVPA min/week	MVPA defined as brisk walking, jogging, running, bicycling, tennis, swimming, other aerobic exercise, other vigorous exercise and stair climbing (>3 METs) and categorised into quintiles according to average min/week:<br>• none (0)<br>• 1–29 (1.1)<br>• 30–59 (3.4)<br>• 60–150 (7.9)<br>• >150 (14.6)	RR for T2D relative to most inactive group:<br>1.00<br>0.84 (0.73, 0.97)<br>0.76 (0.66, 0.88)<br>0.68 (0.60, 0.77)<br>0.48 (0.42, 0.54)<br><br>BMI-adjusted RR for T2D relative to most inactive group:<br>1.00<br>0.94 (0.81, 1.09)<br>0.88 (0.76, 1.02)<br>0.85 (0.74, 0.96)<br>0.66 (0.58, 0.75)		Race, alcohol, weight training, coffee intake, smoking, postmenopausal hormone use, oral contraceptive use, menopausal status, family history of diabetes, total calorie intake, saturated to polyunsaturated fat ratio, trans fat, cereal fibre, wholegrains, glycaemic load (+ BMI in the BMI-adjusted model)
Grøntved et al, 2014 (2)	USA; Nurses' Health Study II (2001–2009) (II)	47,674	F	36–55	8	2.8 (1333/47,674)	The NHS II Physical Activity & Inactivity Questionnaire with biannual postal questionnaires[b]	MVPA min/week	MVPA defined as brisk walking, jogging, running, bicycling, tennis, swimming, other aerobic exercise, other vigorous exercise, and stair climbing (>3 METs) and categorised into quintiles according to average min/week:<br>• none (0)<br>• 1–29 (1.1)<br>• 30–59 (3.4)<br>• 60–50 (7.9)<br>• >150 (14.6)	RR for T2D relative to most inactive group:<br>1.00<br>0.80 (0.67, 0.95)<br>0.68 (0.57, 0.82)<br>0.63 (0.54, 0.74)<br>0.42 (0.36, 0.50)<br><br>BMI-adjusted RR for T2D relative to most inactive group:<br>1.00<br>0.94 (0.79, 1.13)<br>0.83 (0.69, 1.00)<br>0.86 (0.73, 1.01)		Race, alcohol, weight training, coffee, smoking, post-menopausal hormone use, oral contraceptive use, menopausal status, family history of diabetes, total calorie intake, saturated to polyunsaturated fat ratio, trans fat, cereal fibre, wholegrains,

Study	Country; study name	Cohort size	Sex	Age at baseline (years)	Follow-up (years)	% Cumulative incidence (cases/cohort)	Method of PA assessment	PA unit	PA assessment (PA dose in MET h/week[a])	Reported OR/RR/HR (95% CI)	Comments	Adjustments
										0.70 (0.59, 0.83)		glycaemic load (+ BMI in the BMI-adjusted model)
Ding et al, 2015 (23)	Australia; 45 and Up study	54,997	M/F	≥45	3.4	1.6 (888/54,997)	Active Australia survey	PA calculated as the sum of time spent in walking, MVPA and VPA (weighted by a factor of two) in the previous week	Total min MVPA/week:<br>• <150 min (5.6)<br>• 150–<300 min (16.9)<br>• ≥300 min (28.1)	OR for T2D relative to most inactive group:<br>1.00<br>0.72 (0.56, 0.94)<br>0.71 (0.85, 0.97)	Risk estimates were reversed to set the lowest level of LTPA as the referent category using the Hamling method (35)	Age, sex, BMI, SES, health status, BP, blood cholesterol, weight, family history of T2D/heart disease, smoking, alcohol, sitting time, sleep, fruit and vegetable intake, psychological distress

[a]Doses were assigned from descriptions identified within the individual studies or from correspondence with study authors. Full details of MET h dose assignment are listed in ESM Table 2, together with the MMET h/week calculations (see ESM)
[b]Denotes PA assessment questionnaires with published validation and/or reproducibility studies
[c]Studies updated with further follow-up data obtained from the authors
[d]Total cohort
[e]Studies included in the sensitivity analysis using variance-weighted least squares regression analysis

(I)/(II) indicate subcohorts with independently reported risk estimates for T2D within the same publication
CCCC: Chin-Shan community cardiovascular cohort study; China MUCA: China Multicenter Collaborative Study of Cardiovascular Epidemiology; CPA, commuting physical activity; CVD, cardiovascular disease; DPA: daily living physical activity; EE, energy expenditure; ELSA: English longitudinal study of ageing; EPIC-InterAct: European Prospective Investigation into Cancer and Nutrition-InterAct; F, Female; FPG, fasting plasma glucose; M, Male; MEC, Multiethnic cohort; MONICA/KORA: Monitoring Trends and Determinants on Cardiovascular Diseases/Cooperative Research in the Region of Augsburg Cohort Study; NHS, Nurses' Health Study; OPA, occupational physical activity; SES, socioeconomic status

ESM Figure 1 Systematic literature review search terms and strategy

#1 *(activity, physical[MeSH Terms]) OR exercise[MeSH Terms]) OR aerobic exercise[MeSH Terms]) OR motor activity[MeSH Terms]) OR walking[MeSH Terms]) OR OR sport[MeSH Terms]) OR physical fitness[MeSH Terms])) OR physical activity[Title/Abstract]) OR exercise[Title/Abstract]*

#2 *(Type 2 diabetes mellitus[MeSH Terms]) OR niddm[MeSH Terms]) OR diabetes[MeSH Terms])) OR diabetes mellitus, type 2[MeSH Terms]) OR diabetes mellitus, type ii[MeSH Terms]) OR disorders, glucose metabolism[MeSH Terms]) OR impaired glucose[Title/Abstract]) OR type 2 diabetes[Title/Abstract]) OR insulin resistance[Title/Abstract]) OR glucose tolerance[Title/Abstract]*

#3 *(inciden*[Title/Abstract]) OR cohort[Title/Abstract]) OR prospective[Title/Abstract]) OR longitudinal[Title/Abstract]) OR prospective studies[MeSH Terms]) OR prospective study[MeSH Terms]) OR cohort study[MeSH Terms]) OR cohort studies[MeSH Terms])*

#1 and #2 and #3

Search terms for the retrieval of studies in Embase were modified and consisted of combinations of: ‘physical activity’, ‘diabetes mellitus type 2’, ‘NIDDM’, ‘noninsulin dependent diabetes’, ‘cohort study ‘, ‘observational study’ or ‘longitudinal study’.

ESM Figure 2 PRISMA flow chart

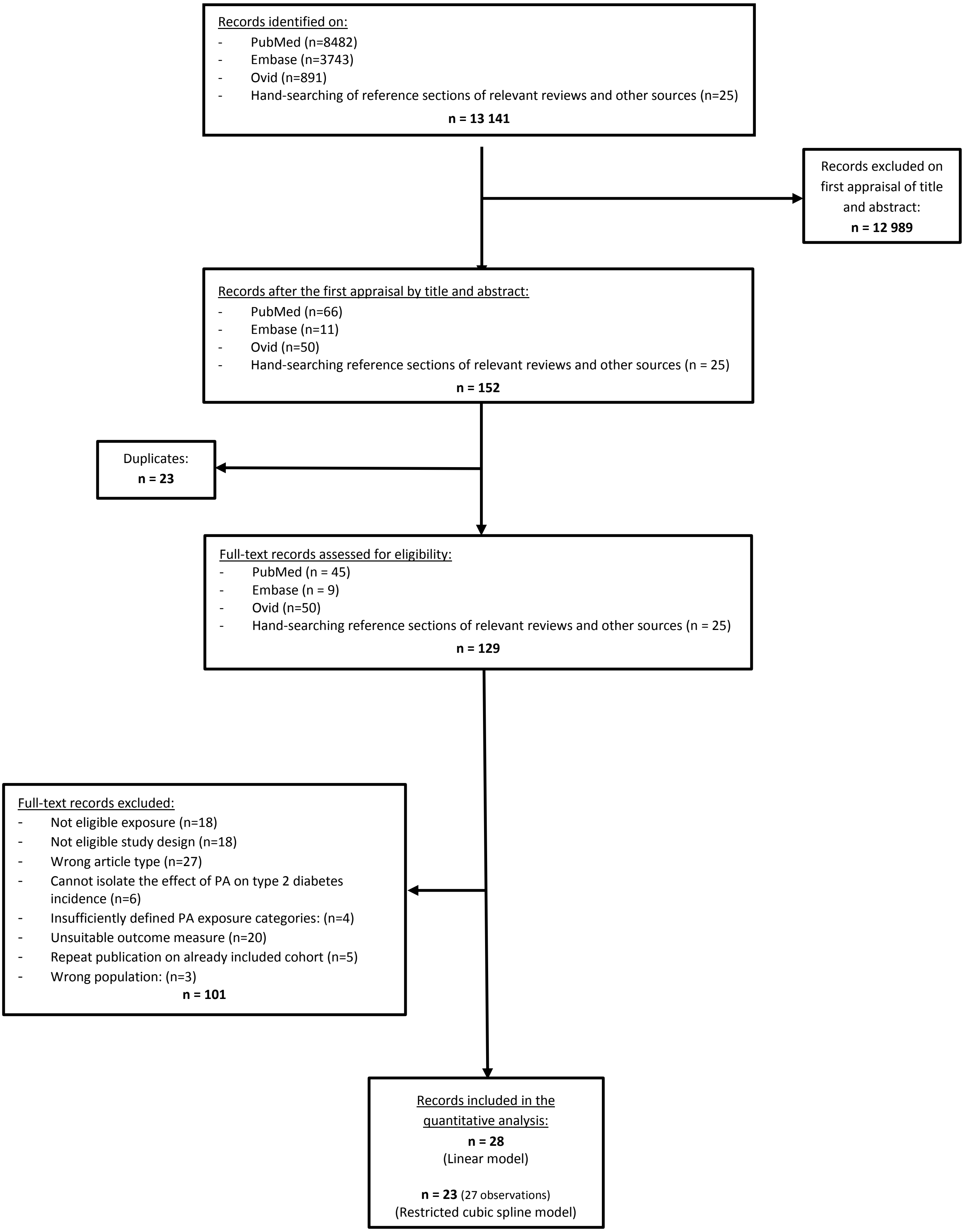

ESM Figure 3 Funnel plot for the linear association of the RR for type 2 diabetes against the standard error of the RR per 10 MET h/week increment of physical activity

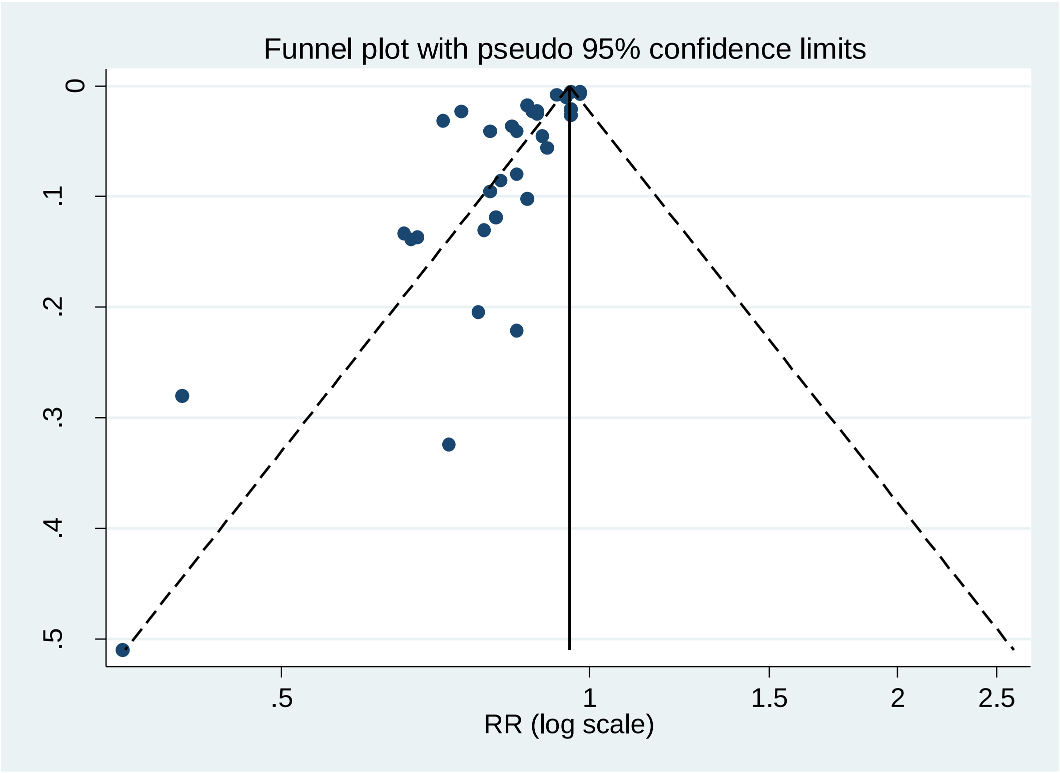

Dashed lines indicate the 95% confidence interval.
Each data point represents one of the 32 observations extracted from 28 cohorts.
Egger's test for publication bias: *p*=0.000

ESM Figure 4 Comparison of the dose-response association and predicted RR point-estimates between LTPA and incidence of diabetes type 2 between the model using restricted cubic splines and the restricted cubic spline model including linear trend estimates of studies not eligible for flexible modelling

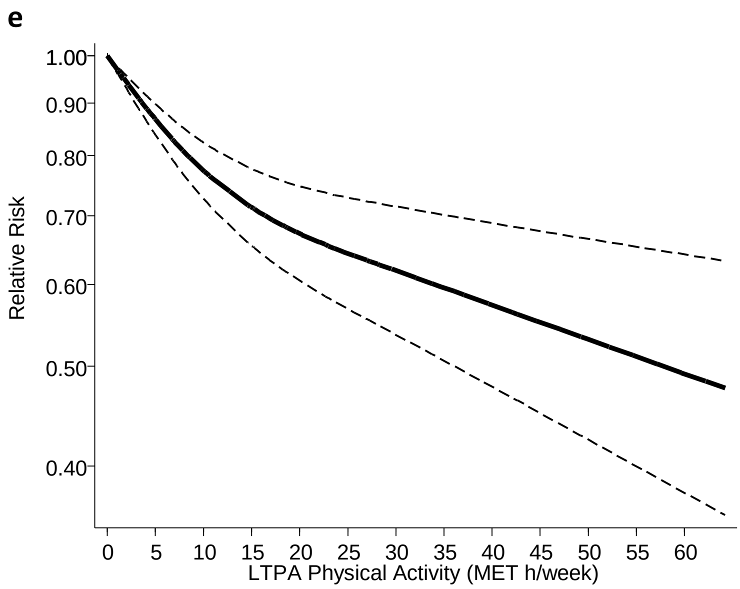

	a		e[1]	
LTPA MET h/week	RR	95% CI	RR	95% CI
2.25	0.93	(0.92, 0.95)	0.94	(0.92, 0.95)
4.50	0.87	(0.84, 0.90)	0.88	(0.85, 0.91)
10.00	0.76	(0.71, 0.81)	0.77	(0.73, 0.82)
11.25	0.74	(0.69, 0.80)	0.76	(0.71, 0.81)
22.50	0.64	(0.56, 0.73)	0.66	(0.58, 0.74)
30.00	0.60	(0.51, 0.70)	0.62	(0.54, 0.72)
60.00	0.47	(0.34, 0.65)	0.50	(0.38, 0.65)

LTPA converted to MET h per week with results pooled in a two stage random effects model. RRs were derived from a common lowest physical activity category within each study. Listed exposure levels were chosen to represent meaningful and easy to interpret PA volumes equivalent to the following: 30 min of MVPA; 1 hour of MVPA; Rounded value to allow for comparison with GLS PA exposure increment; 150 minutes of PA/current recommended guidelines; double the recommended guidelines and two high PA exposure levels investigating the risk reductions at the higher end of the LTPA spectrum. The bold line indicates the pooled restricted cubic spline model and the black dashed line indicates the 95% confidence intervals of the pooled curve.

[1] Model (e) includes additional observations from two independent cohorts that did not report sufficient data to include them in the full model. Sensitivity analysis using variance-weighted least squares (vwls) regression to estimate a linear-within study trend was applied to publications from the Women's Health Initiative (36) and the Shanghai Men's Health Study (37).

Duration assumption was necessary in 9 out of 27 observations and 9 out of 29 observations (applied as 45 min/session in (a) and (e) respectively). Intensity assumption was necessary in 15 out of 27 observations and 17 out of 29 observations (applied as LPA=3 MET, MVPA=4.5 MET, and VPA=8 MET in Scenario (a) and (e) respectively).

References

1. Siegel LC, Sesso HD, Bowman TS, Lee I-M, Manson JE, Gaziano JM. Physical activity, body mass index, and diabetes risk in men: a prospective study. Am J Med. 2009 Dec;122(12):1115–21.

2. Grøntved A, Pan A, Mekary RA, Stampfer M, Willett WC, Manson JE, et al. Muscle-strengthening and conditioning activities and risk of type 2 diabetes: a prospective study in two cohorts of US women. PLoS Med. Public Library of Science; 2014 Jan 14;11(1):e1001587.

3. Wannamethee SG, Shaper AG, Alberti KG, Investigation O. Physical activity, metabolic factors, and the incidence of coronary heart disease and type 2 diabetes. Arch Intern Med. 2000 Jul;160(14):2108–16.

4. Weinstein AR, Sesso HD, Lee IM, Cook NR, Manson JE, Buring JE, et al. Relationship of physical activity vs body mass index with type 2 diabetes in women. J Am Med Assoc [Internet]. 2004 Sep;292(10):1188–94.

5. Hu G, Lindström J, Valle TT, Eriksson JG, Jousilahti P, Silventoinen K, et al. Physical activity, body mass index, and risk of type 2 diabetes in patients with normal or impaired glucose regulation. Arch Intern Med. 2004 Apr 26;164(8):892–6.

6. Folsom AR, Kushi LH, Hong CP. Physical activity and incident diabetes mellitus in postmenopausal women. Am J Public Health. 2000 Jan;90(1):134–8.

7. Helmrich SP, Ragland DR, Leung RW, Paffenbarger RS. Physical activity and reduced occurrence of non-insulin-dependent diabetes mellitus. N Engl J Med. 1991 Jul;325(3):147–52.

8. Haapanen N, Miilunpalo S, Vuori I, Oja P, Pasanen M. Association of leisure time physical activity with the risk of coronary heart disease, hypertension and diabetes in middle-aged men and women. Int J Epidemiol. 1997 Aug;26(4):739–47.

9. Meisinger C, Löwel H, Thorand B, Döring A. Leisure time physical activity and the risk of type 2 diabetes in men and women from the general population. The MONICA/KORA Augsburg Cohort Study. Diabetologia. 2005 Jan;48(1):27–34.

10. Hu FB, Leitzmann MF, Stampfer MJ, Colditz GA, Willett WC, Rimm EB. Physical activity and television watching in relation to risk for type 2 diabetes mellitus in men. Arch Intern Med. 2001 Jun;161(12):1542–8.

11. Okada K, Hayashi T, Tsumura K, Suematsu C, Endo G, Fujii S. Leisure-time physical activity at weekends and the risk of Type 2 diabetes mellitus in Japanese men: the Osaka Health Survey. Diabet Med. 2000 Jan;17(1):53–8.

12. Villegas R, Shu XO, Li H, Yang G, Matthews CE, Leitzmann M, et al. Physical activity and the incidence of type 2 diabetes in the Shanghai women's health study. Int J Epidemiol. 2006 Dec;35(6):1553–62.

13. Lynch J, Helmrich SP, Lakka TA, Kaplan GA, Cohen RD, Salonen R, et al. Moderately intense physical activities and high levels of cardiorespiratory fitness reduce the risk of non-insulin-dependent diabetes mellitus in middle-aged men. Arch Intern Med. 1996 Jun;156(12):1307–14.

14. James SA, Jamjoum L, Raghunathan TE, Strogatz DS, Furth ED, Khazanie PG. Physical activity and NIDDM in African-Americans. The Pitt County Study. Diabetes Care. 1998 Apr;21(4):555–62.

15. Fretts AM, Howard B V, Kriska AM, Smith NL, Lumley T, Lee ET, et al. Physical activity and incident diabetes in American Indians. Am J Epidemiol. 2009 Oct;170(5):632–9.

16. Demakakos P, Hamer M, Stamatakis E, Steptoe A. Low-intensity physical activity is associated with reduced risk of incident type 2 diabetes in older adults: evidence from the English Longitudinal Study of Ageing. Diabetologia. 2010 Sep;53(9):1877–85.

17. Lee D, Park I, Jun T-W, Nam B-H, Cho S, Blair SN, et al. Physical activity and body mass index and their associations with the development of type 2 diabetes in korean men. Am J Epidemiol. 2012 Jul;176(1):43–51.

18. Steinbrecher A, Erber E, Grandinetti A, Nigg C, Kolonel LN, Maskarinec G. Physical activity and risk of type 2 diabetes among Native Hawaiians, Japanese Americans, and Caucasians: the Multiethnic Cohort. J Phys Act Health. 2012 Jul;9(5):634–41.

19. Chien K-L, Chen M-F, Hsu H-C, Su T-C, Lee Y-T. Sports activity and risk of type 2 diabetes in Chinese. Diabetes Res Clin Pract. 2009 Jun;84(3):311–8.

20. Krishnan S, Rosenberg L, Palmer JR. Physical activity and television watching in relation to risk of type 2 diabetes: the Black Women's Health Study. Am J Epidemiol. 2009 Feb;169(4):428–34.

21. Magliano DJ, Barr ELM, Zimmet PZ, Cameron AJ, Dunstan DW, Colagiuri S, et al. Glucose indices, health behaviors, and incidence of diabetes in Australia: the Australian Diabetes, Obesity and Lifestyle Study. Diabetes Care. 2008 Feb;31(2):267–72.

22. Carlsson S, Midthjell K, Tesfamarian MY, Grill V. Age, overweight and physical inactivity increase the risk of latent autoimmune diabetes in adults: results from the Nord-Trøndelag health study. Diabetologia.2007 Jan;50(1):55–8.

23. Ding D, Chong S, Jalaludin B, Comino E, Bauman AE. Risk factors of incident type 2-diabetes mellitus over a 3-year follow-up: Results from a large Australian sample. Diabetes Res Clin Pract. 2015 May;108(2):306–15.

24. Burchfiel CM, Sharp DS, Curb JD, Rodriguez BL, Hwang L-JJ, Marcus EB, et al. Physical Activity and Incidence of Diabetes: The Honolulu Heart Program. Am J Epidemiol. 1995 Feb 15;141(4):360–8.

25. Interact Consortium. Physical activity reduces the risk of incident type 2 diabetes in general and in abdominally lean and obese men and women: The EPIC-InterAct study. Diabetologia. 2012 Jul;55(7):1944–52.

26. Fan S, Chen J, Huang J, Li Y, Zhao L, Liu X, et al. Physical activity level and incident type 2 diabetes among chinese adults. Med Sci Sports Exerc. 2015 Aug;47(4):751–6.

27. Nakanishi N, Takatorige T, Suzuki K. Daily life activity and risk of developing impaired fasting glucose or type 2 diabetes in middle-aged Japanese men. Diabetologia. 2004 Oct;47(10):1768–75.

28. Manson JE, Nathan DM, Krolewski AS, Stampfer MJ, Willett WC, Hennekens CH. A prospective study of exercise and incidence of diabetes among US male physicians. JAMA. 1992 Jul;268(1):63–7.

29. Hu FB, Manson JE, Stampfer MJ, Colditz G, Liu S, Solomon CG, et al. Diet, lifestyle, and the risk of type 2 diabetes mellitus in women. N Engl J Med. 2001 Sep;345(11):790–7.

30. Stoudt HW, Damon A, McFarland R, Roberts J. Weight, height, and selected body dimensions of adults, united states-1960-1962. Vital Health Stat 11. 1965 Jun;11:1–44.

31. Prättälä R, Sippola R, Lahti-Koski M, Laaksonen MT, Mäkinen T, Roos E. Twenty-five year trends in body mass index by education and income in Finland. BMC Public Health. 2012 Jan;12(1):936.

32. Peters T, Brage S, Westgate K, Franks PW, Gradmark A, Tormo Diaz MJ, et al. Validity of a short questionnaire to assess physical activity in 10 European countries. Eur J Epidemiol. 2012 Jan;27(1):15–25.

33. Walpole SC, Prieto-Merino D, Edwards P, Cleland J, Stevens G, Roberts I. The weight of nations: an estimation of adult human biomass. BMC Public Health. 2012 Jan; 12:439.

34. Grøntved A, Rimm EB, Willett WC, Andersen LB, Hu FB. A prospective study of weight training and risk of type 2 diabetes mellitus in men. Arch Intern Med. 2012 Oct;172(17):1306–12.

35. Hamling J, Lee P, Weitkunat R, Ambühl M. Facilitating meta-analyses by deriving relative effect and precision estimates for alternative comparisons from a set of estimates presented by exposure level or disease category. Stat Med. 2008 Mar;27(7):954–70.

36. Hsia J, Wu L, Allen C, Oberman A, Lawson WE, Torréns J, et al. Physical activity and diabetes risk in postmenopausal women. Am J Prev Med. 2005 Jan;28(1):19–25.

37. Shi L, Shu X-O, Li H, Cai H, Liu Q, Zheng W, et al. Physical activity, smoking, and alcohol consumption in association with incidence of type 2 diabetes among middle-aged and elderly Chinese men. PLoS One. 2013 Jan;8(11):e77919.

38. Food and Agriculture Organization of the United Nations. Human energy requirements - Principles and definitions. 2004.
